# Hybrid Cellulosic Substrates Impregnated with Meta-PBI-Stabilized Carbon Nanotubes/Plant Extract-Synthesized Zinc Oxide—Antibacterial and Photocatalytic Dye Degradation Study

**DOI:** 10.3390/nano14161346

**Published:** 2024-08-14

**Authors:** Hristo Penchev, Katerina Zaharieva, Silvia Dimova, Georgy Grancharov, Petar D. Petrov, Maria Shipochka, Ognian Dimitrov, Irina Lazarkevich, Stephan Engibarov, Rumyana Eneva

**Affiliations:** 1 Institute of Polymers, Bulgarian Academy of Sciences, “Akad. G. Bonchev” St., Block 103A, 1113 Sofia, Bulgaria; dimova@polymer.bas.bg (S.D.); granchar@polymer.bas.bg (G.G.); ppetrov@polymer.bas.bg (P.D.P.); 2 Institute of Mineralogy and Crystallography, “Acad. I. Kostov”, Bulgarian Academy of Sciences, Acad. G. Bonchev St., Block 107, 1113 Sofia, Bulgaria; 3 Institute of General and Inorganic Chemistry, Bulgarian Academy of Sciences, “Acad. G. Bonchev” St., Bl. 11, 1113 Sofia, Bulgaria; shipochka@svr.igic.bas.bg; 4 Institute of Electrochemistry and Energy Systems, “Acad. Evgeni Budevski”, Bulgarian Academy of Sciences, Acad. G. Bonchev St., Block 10, 1113 Sofia, Bulgaria; ognian.dimitrov@iees.bas.bg; 5 The Stephan Angeloff Institute of Microbiology, “Acad. G. Bonchev” St., Block 26, 1113 Sofia, Bulgaria; irinalazarkevich@abv.bg (I.L.); pprox_engibarov@abv.bg (S.E.); rum_eneva@abv.bg (R.E.)

**Keywords:** meta-polybenzimidazole, carbon nanotubes, zinc oxide, cellulose, hybrid material, green synthesis, photocatalyst, methylene blue, antibacterial

## Abstract

Novel fibrous cellulosic substrates impregnated with meta-polybenzimidazole (PBI)-stabilized carbon nanotubes/zinc oxide with different weight content of ZnO and with the use of dimethylacetamide as dispersant media. The pristine ZnO nanoparticle powder was prepared by plant extract-mediated synthesis using *Vaccinium vitis-idaea* L. The green synthesized ZnO possesses an average crystallite size of 15 nm. The formation of agglomerates from ZnO NPs with size 250 nm–350 nm in the m-PBI@CNTs/ZnO was determined. The prepared materials were investigated by PXRD analysis, XPS, SEM, EDS, AFM, and TEM in order to establish the phase and surface composition, structure, and morphology of the hybrids. The potential of the synthesized hybrid composites to degrade methylene blue (MB) dye as a model contaminant in aqueous solutions under UV illumination was studied. The photocatalytic results show that in the course of the photocatalytic reaction, the m-PBI@CNTs/ZnO 1:3 photocatalyst leads to the highest degree of degradation of the methylene blue dye (67%) in comparison with the other two studied m-PBI@CNTs/ZnO 1:1 and 1:2 composites (48% and 41%). The antibacterial activity of ZnO nanoparticles and the hybrid CNT materials was evaluated by the RMDA and the dynamic contact method, respectively. The profound antibacterial effect of the m-PBI@CNTs/ZnO hybrids was monitored for 120 h of exposition in dark and UV illumination regimes. The photocatalytic property of ZnO nanoparticles significantly shortens the time for bactericidal action of the composites in both regimes. The m-PBI@CNTs/ZnO 1:2 combination achieved complete elimination of 5.10^5^ CFU/mL *E. coli* cells after 10 min of UV irradiation.

## 1. Introduction

Dyes mainly released from the textile and dyeing industries have a significant impact on the environment by destroying life forms, absorbing the dissolved oxygen, and changing the pH and temperature profile of the aquatic environment [1]. One of the most commonly used dyes in industries, medicine, and chemical activities is Methylene Blue (MB), which is a heterocyclic organic compound with a phenothiazine structure. Water contamination with MB causes serious health problems such as blindness, respiratory disorders, and digestive diseases. In addition, the presence of MB in water, even at extremely low concentrations, impacts photosynthesis by reducing sunlight penetration [2]. The photocatalytic process has gained attraction due to its ability to degrade pollutants in the presence of different light sources. Zinc oxide (ZnO) is regarded as a multifunctional material. It is one of the widely studied semiconductor materials with various applications. ZnO is used in photocatalytic applications under UV–vis light irradiation due to the wide band gap of 3.37 eV and high exciton binding energy of 60 meV at room temperature. It has attracted attention due to the presence of unique properties (electrical, optoelectronic, and luminescent) as well as the ability to degrade various pollutants, processing non-toxic nature, and its relatively low cost/widely available [1]. In numerous studies, ZnO in nanoparticle form (NPs) has shown greater photocatalytic activity than its main rival—titanium dioxide, especially in organic dye degradation [3,4]. As a particular drawback, ZnO is characterized by the relatively high rate of charge carriers’ recombination [5], an intimate tendency for aggregation as well as diminished photo stability under prolonged UV and sunlight irradiation [6]. The rate of charge carriers recombination is dependent on the size of the ZnO NPs [7]. This limits its use in advance oxidation process (AOP) wastewater treatment. ZnO NPs process high surface energy, which induces agglomeration and naturally leads to deterioration of the photocatalytic efficiency of ZnO by greatly reducing their effectively developed surface [8]. Therefore, the synergy effect rendered by coupling ZnO with carbonaceous nanomaterial such as carbon nanotubes (CNTs) can obviate the drawbacks of the sole ZnO photocatalyst. This is because CNTs can retard the recombination rate of photogenerated electron and hole pairs by consistently accepting the electron from ZnO [9]. Additionally, suitable chemical stability and corrosion resistance of the CNTs can suppress the photocorrosion of ZnO and thus enhance the photocatalytic efficiency concomitantly [6]. Moreover, the high aspect ratio and large specific surface area of CNTs allow the adhesion of ZnO on its surface to avoid the formation of aggregates [10].

Because of their unique physical and chemical properties, carbon nanotubes (CNTs) are considered to be excellent candidates for many potential applications, such as nanocomposite materials, nanoelectronics, catalysis, and sensors [11]. Due to their great individual toughness in the gigapascal (Gpa) range, both single- and multiwall CNTs (MWCNTs) keep their structure and integrity even at high nanoparticle loadings in a great variety of polymer composite and organic–inorganic hybrid formulations [9,12]. Recently, CNTs have been used as templates or scaffolds for the hybrid assembly of nanoparticles and are widely reported to synergistically enhance the photocatalytic activity of metal oxide nanoparticles through the retardation of electron–hole transfer and recombination [13].

Different metal oxides such as ZnO, TiO_2_, Fe_3_O_4_, ZrO_2_, SnO_2_, MnO_2_, Ta_2_O_5_, and Nb_2_O_5_ could provide enhanced photocatalytic activity even at lower percentages of CNTs [14]. Various CNTsT/ZnO composites are used in versatile applications such as photocatalysis, optics, coatings, drug delivery, gene delivery, photonics, and microelectronics [15]. This feature is attributed to the synergistic effects of supporting and electron–hole recombination and electron trapping effect on the transition metals or semiconductive metal oxides surface. The CNTs’ inorganic hybrid materials showed not only better activity as unsupported materials but also they can be easily recovered and reused as fresh catalysts [16]. 

Polybenzimidazoles (PBIs) are a class of non-flammable, heat-resistant, mechanically robust, and chemically resistant heterocyclic thermoset polymers [17]. The incorporation of different nanofillers, including MWCNTs, into the PBI matrix has motivated extensive research progress in the field of high-performance nanocomposites [18]. Nanocomposites of thermally stable PBI containing small amounts (<1%) of functionalized MWCNTs were prepared using solution casting methods and investigated for alkaline direct methanol fuel cell application [19].

Cellulose is the most abundant biopolymer in nature and is known for its distinguished properties, such as being renewable, biodegradable, cheap, and widely available in different pristine and man-made formulations. Surface impregnating of fibrous cellulose substrates with other nano- and nanocomposite materials boost the properties like microporosity, hydrophilicity, and their specific surface area. Cellulose-based scaffolds are environmentally friendly and sustainable, which justifies the requirements of a suitable photocatalyst by enhancing the adsorption ability and degradation efficiency of the hybrid photocatalyst substrates therefrom [20]. 

The green or biological approaches for the preparation of nanosized ZnO have been considered due to being safe, clean, cost-effective, and the least toxic emission [21]. The synthesis of metal oxide nanoparticles with the use of plant extract is a promising alternative to the conventional chemical method [22]. For example, green ZnO NPs were synthesized using plant leaf extract (*Aloe barbadensis Mill*) [23] and other kinds of plant extracts [24]. To our knowledge, plant extract of lingonberry (*Vaccinium vitis-idaea* L.). is still not reported to be used in the green synthesis approach of ZnO nanoparticles. 

ZnO/CNTs nanocomposites were successfully synthesized by the laser ablation process [25], ultrasonication/hydrothermal route [26], and modified sol–gel method [27] via a simple step-by-step chemical synthesis process, followed by a co-crystallization approach [28] and demonstrate suitable catalytic activity against MB dye. A series of ZnO–single-walled CNTs nanocomposite photocatalysts were synthesized for the decomposition of MB [29]. Also, ZnO/CNTs nanocomposites were synthesized by microwave-assisted hydrothermal methods and showed high adsorption capacity in removing the Brilliant Green [30]. The MWCNTs/ZnO nanocomposite was synthesized using the sol–gel process and investigated for photocatalytic decolorization of crystal violate dye [31]. The fabrication of carbon nanotubes/cellulose composite paper fabricated via vacuum filtration of sodium dodecylsulfate-stabilized CNTs with cellulose pulp was also reported by Pang et al. [32], but direct impregnation of disposable cellulosic substrate with PBI-stabilized CNTs and mixed CNTs/ZnO dispersions is reported for the first time in the present work. 

The antimicrobial properties of ZnO are investigated and described in a number of publications and review articles [33,34,35]. ZnO NPs act on cells by producing reactive oxygen species (ROS)—highly active forms of molecular oxygen like O_2_^•−^ (superoxide anion), OH^•^ (hydroxyl radical), H_2_O_2_ (hydrogen peroxide), ^1^O_2_ (singlet oxygen), and HOCl (hypochlorous acid). ROS are generated intrinsically by the cell or by factors in its environment [34]. They oxidize various types of macromolecules in the cell. Thus, they cause damage to virtually all cellular processes by one basic mechanism, namely the slow release of bacteriostatic zinc ions from the particle surface and the direct ZnO nanoparticle interactions with the microbial cell surface and the cytoplasm [36,37]. 

In the present work, for the first time, meta-polybenzimidazole-stabilized CNTs dispersion, as well as mixed dispersions containing plant extract-synthesized ZnO NPs, were evenly deposited onto a microfibrous cellulosic substrate, and the resultant hybrid impregnated scaffolds were preliminarily tested for their photocatalytic methylene blue dye degradation and for their antimicrobial activity toward two bacterial strains. The effect of ZnO NPs loading on both the photocatalytic and antibacterial activity was elucidated under UV irradiation and in the dark.

## 2. Materials and Methods

### 2.1. Materials

N,N-Dimethylacetamide (p.a., ≥99.5% (GC)) as a solvent, commercial S-26 meta-polybenzimidazole solution, 0.7 dL/g (PBI Performance Products, Inc., Charlotte, NC, USA), Timestub^TM^-Graphitized Multi-walled Carbon nanotubes, TNGM3, purity: >99%, OD: 10–20 nm, length: 5–30 µm, SSA: >80 m^2^/g, Zn(NO_3_)_2_·6H_2_O p.a. ≥ 98%, methylene blue p.a. (Valerus Co.), NaOH, KOH technical grade (Valerus Co.), ethanol 96% and *Vaccinium vitis-idaea* L. (dried leaves) from local supplier, and cellulosic filter paper Boeco 389 class with typical pore size: 8–12 µm were obtained.

### 2.2. Green Synthesis of Zinc Oxide Nanoparticles

Zinc oxide nanoparticles were synthesized using 0.18M Zn(NO_3_)_2_·6H_2_O (Valerus Co.) and 2M NaHCO_3_ (Valerus Co.) aqueous solutions. The two solutions were separately stirred for 10 min. The novel plant extract (15 mL) of *Vaccinium vitis-idaea* L. was added to the zinc nitrate aqueous solution and stirred for 30 min. The plant extract was prepared using 6.022 g dried leaves of *Vaccinium vitis-idaea* L. in 200 mL distilled water at stirring for 1 h at 75 °C. The hot mixture stood for 30 min and, after that, was filtered. The precipitant NaHCO_3_ was added dropwise to the mixture of zinc nitrate and plant extract at continuous stirring until reaching pH 7, respectively. The suspension was stirred for two hours after precipitation. The precipitate was filtered and washed with distilled water several times. The obtained precipitate was dried at 50 °C and thermally treated at 370 °C for 2 h in air media.

### 2.3. Preparation of Polybenzimidazole-Stabilized Multi-Walled Carbon Nanotubes/Zinc Oxide Hybrid Dispersions

Regenerated m-PBI powder, purified from LiCl salt solvent additive and from oligomeric PBI fractions, was obtained by precipitated ethanolic/KOH PBI solution as previously described [38]. Mixing a dilute solution of regenerated m-PBI and MWCNTs with or without ZnO nanoparticles in dimethylacetamide (DMAc) as polar aprotic solvent afforded stable PBI-wrapped MWCNTs (denoted as PBI@CNTs) and mixed MWCNTs/ZnO dispersions (denoted PBI@CNTs/ZnO). Two kinds of dispersions were prepared, namely, single PBI@CNTs and mixed PBI@CNTs/ZnO with three different weight ratios of MWCNTs to ZnO 1:1, 1:2, and 1:3. In a typical synthetic procedure, 10 mg of purified m-PBI was dissolved in 20 mL of DMAc, and then 20 mg of MWCNTs were added. After that, a short 30 s ultrasonication treatment was applied until completely stable dark black dispersion with a concentration of MWCNTs of 1 mg/mL was obtained. 

For the preparation of mixed PBI@CNTs/ZnO dispersions, the dry green synthesized ZnO powder was directly ultrasound treatment introduced into the stock PBI@CNTs dispersion by mixing at three different weight ratios according to the CNTs content. 

### 2.4. Surface Impregnation of Cellulosic Filter Paper Substrate with PBI@CNTs and PBI@CNTs/ZnO Dispersions

Free drop spreading method was used for deposition and impregnation of cellulosic filter paper substrates (ϕ 9 cm) with 3 mL of both bare PBI@CNTs and the three different mixed PBI@CNTs/ZnO dispersions with the above-mentioned content and concentration characteristics. 

### 2.5. Physicochemical Characterization of Prepared PBI-Stabilized Carbon Nanotubes/Green Synthesized ZnO Composites 

The powder X-ray diffraction (PXRD) analysis was carried out on an X-ray powder diffractometer “Empyrean” within the range of 2θ values between 3° and 100° using Cu Kα radiation (λ = 0.154060 nm) at 40 kV and 30 mA. The identification of phases was performed through High Score Plus, Version 4.9 (4.9.0.27512) software. The mean crystallite size (D), lattice strain (ε), and unite cell parameter (a) of the zinc oxide phase were determined using the PowderCell, Version 2.4 software [39] and using the Williamson–Hall equation [40]:

*β* cos *θ* = 0.9 *λ*/*D* + 4*ε* sin *θ* (1)

where ε is the value of internal strain, β is the full-width half-maximum (FWHM) of diffraction, θ is the Bragg’s angle, λ is the wavelength of the X-ray beam used, and D is the average crystallite size of the phase under study.

The film composition and electronic structure were investigated by X-ray photoelectron spectroscopy (XPS). The measurements were carried out on an AXIS Supra electron- spectrometer (Kratos Analytical Ltd., Manchester, UK) using monochromatic AlKα radiation with a photon energy of 1486.6 eV and a charge neutralization system. The binding energies (Bes) were determined with an accuracy of ±0.1 eV. The chemical composition in the depth of the films was determined by monitoring the areas and binding energies of C1s, O1s, N1s, and Zn2p photoelectron peaks. Using the commercial data-processing software of Kratos Analytical Ltd., the concentrations of the different chemical elements (in atomic %) were calculated by normalizing the areas of the photoelectron peaks to their relative sensitivity factors.

Attenuated total reflection Fourier transform infrared (ATR-FTIR) spectroscopic analysis was performed on an IR Affinity-1 spectrophotometer (Shimadzu, Kyoto, Japan) equipped with a MIRacle ATR accessory (diamond crystal, depth of penetration of the IR beam into the material is 2 μm). 

The surface morphology of the samples was investigated with scanning electron microscopy (SEM) using a Zeiss Evo 10 microscope (Carl Zeiss Microscopy, Oberkochen, Germany). The photographs were taken in secondary electrons and backscattered electrons mode with an accelerating voltage of 25 keV and no conductive coating on the samples. The chemical composition of the surface was studied with electron dispersive spectroscopy (EDS) probe Oxford Ultim Max 40 (Oxford Instruments, Abingdon, United Kingdom). The results were compiled with Aztec software (version 6.1 HF4). The statistical measurements of carbon nanotubes were performed with Image J 1.53t software.

TEM analyses were carried out on an HR STEM JEOL JEM 2100 (JEOL Ltd., Tokyo, Japan).

AFM imaging was performed on a Bruker Dimension Icon microscope working under Peak Force Tapping (PFT) mode. The micrographs were recorded at room temperature using silicon nitride tips with a resonance frequency of approximately 70 kHz and a spring constant of 0.4 N/m at the scan rate of 1.0 Hz

The hydrodynamic diameter and size distribution of the hybrids were determined with a Zetasizer NanoBrook 90Plus PALS instrument (Brookhaven Instruments Corporation, Holtsville, NY, USA) equipped with a 35 mW red diode laser (λ = 640 nm) at a scattering angle of 90°. The sample concentration was 1.0 g L^−1^, and 3 measurements were performed for each sample at a temperature of 25 °C.

### 2.6. Photocatalytic Tests 

The photocatalytic degradation of Methylene Blue dye as a model pollutant with an initial concentration of aqueous solution of the dye—5 ppm has been investigated. The prepared PBI-stabilized carbon nanotubes/green synthesized ZnO composites as photocatalysts were used. The UV-A illumination lamp with maximum emission at 365 nm; 18 W and illumination intensity 2.6 mW/cm^2^ was used. The UV irradiation dose for 180 min of exposure was 3.921 × 10^−17^ J/m^2^. The photocatalytic investigations were carried out without and in the presence of 0.015 M solution of H_2_O_2_. The photocatalytic experiments were performed in a semi-batch slurry reactor equipped with two frits blowing tiny bubbles of air in order to saturate the solution in dissolved oxygen using 3 cm × 3 cm impregnated cellulose substrate photocatalyst and 20 mL of dye solution under a constant stirring rate (400 rpm). In the tested systems, the dye solution and photocatalyst were stirred in the dark for about 30 min before switching on the UV irradiation for 3 h in order to reach adsorption–desorption equilibrium state. The investigations of the photocatalytic abilities of synthesized hybrid materials were carried out by taking aliquot samples of the suspension out of the reaction vessel after regular time intervals. The reaction course was monitored by UV–vis absorbance spectrophotometer UV-1600PC in the wavelength range from 200 to 800 nm (λmax = 664 nm for MB). The degree of degradation is determined using dependence: ((C_0_ – C)/C_0_) × 100, where C_0_ and C are, respectively, the initial concentration before turning on the illumination and the residual concentration of the dye solution after illumination in the course of a given time interval. 

### 2.7. Antibacterial Activity

#### 2.7.1. Media and Test Microorganisms

Mueller–Hinton Broth (MHB) and Mueller–Hinton Agar (MHA) (HiMedia Laboratories, Mumbai, India) were used for the preparation of standardized bacterial cell suspensions and the cell number counting. Samples of the obtained materials, including powders of ZnO nanoparticles and MWCNTs, were autoclaved at 120 °C for 10 min. All experiments were performed with pre-sterilized samples. The following test microorganisms were used: *Escherichia coli* ATCC 25922 and *Bacillus subtilis* ATCC 6633.

#### 2.7.2. Antibacterial Properties

Release of Solutes and Ions from the Materials or Their Constituents

Overnight cultures of the test organisms (100 µL) were spread on MHB agar plates. Pieces (0.2 cm^2^) of the materials were placed on the inoculated agar plates. Drops of saline suspension (50 μL, 1 mg/mL) of the ZnO nanoparticles and CNTs were pipetted in wells of the agar in the same dish, and it was incubated for 24 h at 30 °C.

##### Minimum Inhibitory Concentration (MIC) of ZnO and CNT Nanoparticles

MICs were determined by the Resazurin-based Microtitre Dilution Assay (RMDA) as applied by [41]. The starting suspensions had a concentration of 5 mg/mL for ZnO and 10 mg/mL for CNTs in distilled water. They were vortexed and sonicated before use. MIC of CNTs was tested in two variants—static and on a wrist shaker with 120 rpm.

The antimicrobial activity of the hybrid materials and nanopowders were tested according to ASTM Standard Test Method E 2149–10 [42], adapted for a 24-well sterile plate. A 0.5 cm^2^ sample of each material was placed in a well and poured with 1 mL of a standardized cell suspension of *E. coli* containing approximately 1.5 ÷ 3.0 × 10^6^ cells in a sterile buffer solution prepared according to the method requirements [43]. Control wells contained only 1 mL of bacterial suspension. Samples and controls were set up in duplicate. The plate was incubated in a thermostat at 30 °C on a wrist-action shaker at 120 rpm to achieve dynamic contact of cells with the sample surface. The number of viable cells in each well was counted at the following intervals: 0, 1 h, 24, 48, and 120 h by the modified Koch method of tenfold serial dilutions and Petri plating.

#### 2.7.3. Photocatalytic Antibacterial Activity of Bare and PBI-Stabilized ZnO Nanoparticles

For the photocatalytic antibacterial test, the same protocol for standardizing the cell suspension of *E. coli* was used. When the optical density was set to ~1 × 10^8^ colony forming units (CFU)/mL, 100 μL of this suspension was transferred into a 50 mL beaker, already containing 9.9 mL of 0.5 mg/mL suspension of ZnO nanoparticles. Thus, we obtained a 10 mL suspension of ~1 × 10^6^ CFU/mL and 0.5 mg/mL ZnO and PBI@ZnO nanoparticles. The next steps of the procedure were performed according to [44]. Briefly, the mixture was divided into two beakers, containing 5 mL each, and stirred on two parallel magnetic stirrer platforms installed in a closed dark box. After 15 min of dark incubation, the photocatalytic reaction was started for one of the beakers by switching on a 15 W UV-A lamp (0.2 A/50 Hz, Medicor, Budapest, Hungary) at a distance of 0.8 m from the suspension surface. The second beaker was covered by an aluminum foil lid to remain in the dark. Bacterial enumeration was carried out as 100 μL of sample aliquots were taken from each beaker at 0, 10, 20, and 30 min after the UV light exposure. The number of viable cells was determined by plating 20 μL from a suitable dilution in a Petri dish and 24 h of incubation at 30 °C. All antibacterial tests were performed in three replicates. 

#### 2.7.4. Photocatalytic Antibacterial Activity of the Hybrid Materials

A 24-well plate was divided into two zones. Pieces of 1 cm^2^ from each sample were placed in symmetrical wells of the two zones. A total of 1 mL of *E. coli* suspension containing 5 × 10^5^ CFU/mL was poured onto them. One control well in both zones contained only bacterial suspension. After standing at room temperature overnight, one of the areas in the plate was irradiated by a UV light source for 10 min, and the other was covered with aluminum foil to remain in the dark. After 1 h, a 20 µL sample was taken from each well and dropped on a sector in a Petri dish with Nutrient agar. The result is read after 24 h of cultivation in a thermostat at 30 °C.

## 3. Results and Discussion

### 3.1. Preparation of PBI-Stabilized CNTs and/orZnO Impregnation Dispersions

**Figure 1:**
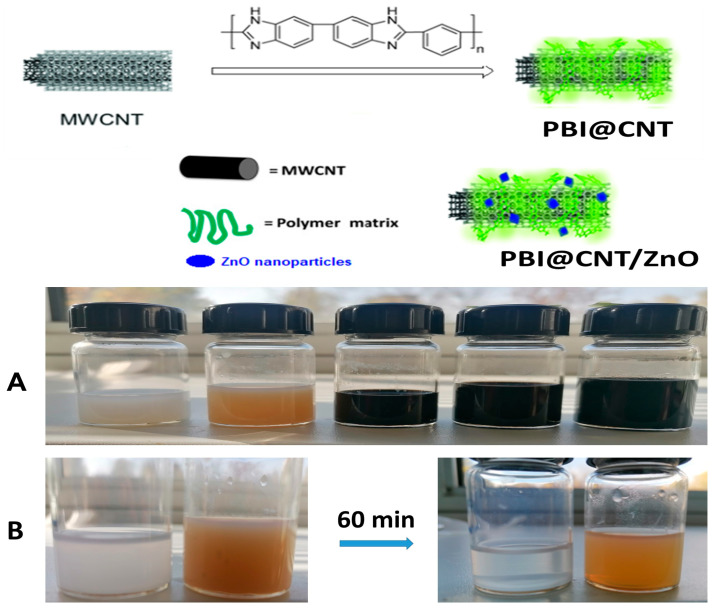
Schematic representation of m-PBI surface wrapping stabilization of MWCNTs. Pictures of the hybrid dispersions from left to right: pristine ZnO NPs dispersed in DMAc; m-PBI@ZnO; m-PBI@CNTs; m-PBI@CNTs/ZnO 1:1 and m-PBI@CNTs/ZnO 3:1 (**A**); time stability comparison of the bare plant extract ZnO NPs and m-PBI@ZnO dispersions in DMAc before (**left**) and after 60 min stay (**right**) (**B**).

In order to obtain highly concentrated and stable/deagglomerated dispersions of otherwise inherently hydro-/organophobic and with highly entangled/agglomerated bulk MWCNTs structure, we used polymer surface wrapping strategy with the use of purified meta-PBI as a surface stabilizer and deagglomerating agent in pure DMAc as aprotic solvent as shown on Figure 1A,B. Our preliminary results with the use of mixed DMAc/LiCl stock solvent solution lead to a partially unsatisfactory result, which is probably due to imidazolic PBI rings H-bonds interruption by the Li-salt present. With the use of a combination of ethanolic KOH regenerated m-PBI and pure DMAc solvent, we achieved highly concentrated and very stable dispersions with a concentration of CNTs up to 5 mg/mL, but later, for practical reasons, we used stock dispersions with a concentration of 1 mg/mL for the cellulose substrate impregnation experiments. We assume that the stabilization was achieved through the spontaneous wrapping of the conjugated PBI macromolecular chains round the MWCNTs surface, which is driven by specific π-π interactions of the PBI skeleton with the aromatic C6 rings from CNTs, similarly observed for AB-PBI/CNTs and Pt-PBI/CNTs composites [45,46]. In our CNT dispersion methodology, we used a very short ultrasonication treatment time of about 30 s in order to avoid the occurrence of unwanted CNT mechano-chemical damage [47,48]. It is interesting to mention that the PBI-wrapped carbon nanotubes from DMAc dispersion were observed as a dense swarm of individual free-moving Brownian motion worm-like particles observed by simple light microscopy at 600× imagination (video presented in Supplementary Materials). The inherent long-term dispersion stability of the as-obtained concentrated colloidal suspensions of PBI-stabilized MWCNTs and the easy preparation procedure makes them, in a wide prospect, potentially valuable for diverse research purposes and for education/demonstration purposes in particular as conventional light microscopy is fully available technique even in primary schools worldwide. Our preliminary experiments for ultrasound dispersion of green synthesized ZnO NPs (1 mg/mL) in bare DMAc solvent without the addition of PBIs resulted in quasi-stable dispersion, which almost completely settled down after 1 h stay as opposed to 1 to 1 wt. ratio PBI containing dispersion, which was stable overnight (Figure 1B). This is an indication of the occurrence of some specific, most probably ionogenic and Zn-coordination interactions between the surface of the ZnO nanophase and the imidazolic rings of PBI, as evidenced by a previous study [49]. 

### 3.2. Characterization of Prepared PBI-Stabilized Carbon Nanotubes/ZnO Dispersions and Impregnated Cellulose Substrates

#### 3.2.1. DLS Measurement of PBI@CNTs and PBI@CNTs/ZnO Dispersions

**Figure 2:**
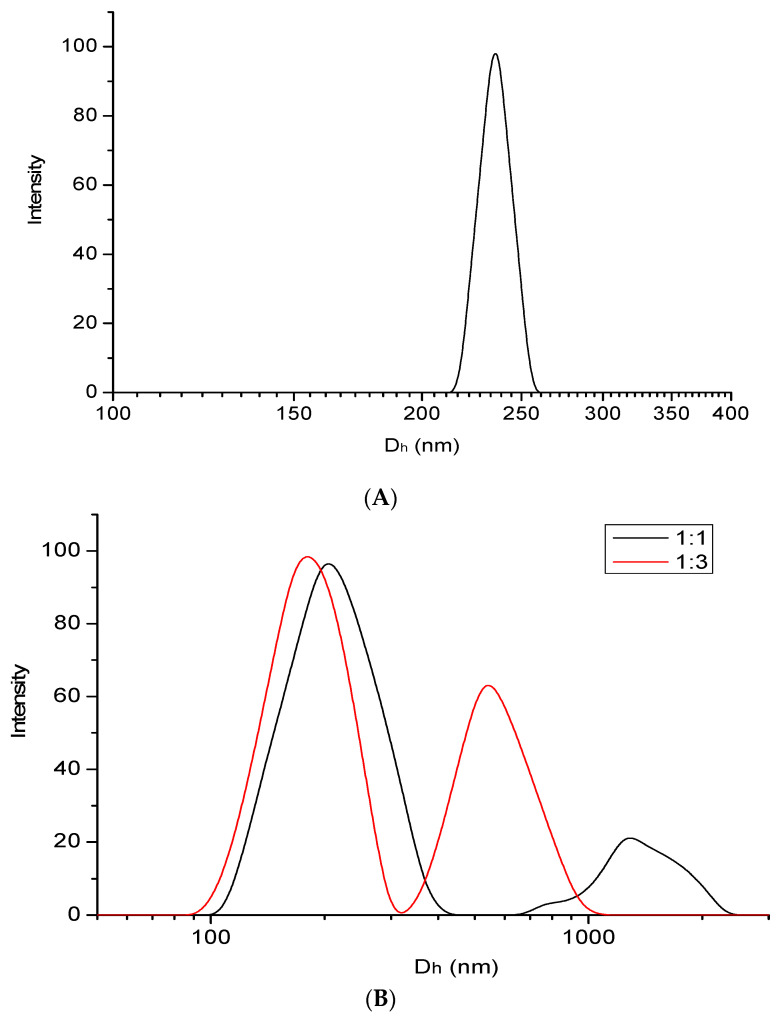
Size distribution plot of PBI-modified MWCNTs dispersions in DMAc (**A**). Size distribution plots of PBI-modified MWCNTs/ZnO dispersions in DMAA at different CNTs/ZnO mass ratios (**B**).

Dynamic light scattering technique was used to evaluate the hydrodynamic diameter and size distribution of both m-PBI@CNTs and m-PBI@CNTs/ZnO dispersions in DMAc solvent. DLS is usually applied to study the size of CNT agglomerates and is thus very well suited to follow the success of the dispersion process [48]. DLS measurement can also provide valuable integral information on the size distribution of CNTs in dispersant media [50]. The results from DLS measurements for m-PBI@CNTs (Figure 2A) showed a monomodal distribution of well-dispersed multi-walled carbon nanotubes with a mean hydrodynamic diameter of 230 nm. Having in mind the given producer’s parameters for mean diameter and average nanotube length of the used bulk MWCNTs (OD: 10–20 nm, length: 5–30 µm), the obtained result seems to be in close approximation with the DLS measurement for single wall CNTs with a mean diameter of 6 nm and avr. Length of 700 nm [51]. The addition of bulk powder agglomerated ZnO NPs into the already formed m-PBI@CNTs dispersion at different weight ratios and short ultrasonication led to the observation of bimodal particle distribution of a mixed CNTs/ZnO fraction at pprox.. 350 nm (for m-PBI@CNTs/ZnO 1:1) and 250 nm (for m-PBI@CNTs/ZnO 1:3). The second population is probably pure ZnO agglomerates fraction with avr. Hydrodynamic diameter of 800 nm for a 1:3 CNTs/ZnO ratio and a minor particle fraction at about 1200 nm for a 1:1 CNTs/ZnO ratio. It seems that with the increase in ZnO feed content in the mixed m-PBI@CNTs dispersions, the sonication process improves the dispersibility and deagglomeration of the ZnO particles phase, which could be explained by the increased release of Zn^2+^ ions and the overall increased ZnO active surface and the specific multimode interactions with the PBI-stabilized CNTs nano-interphase as well as interactions with the small fraction of free PBI molecules in the solution. The polydispersity index of the PBI@CNTs sample is 0.12 ± 0.02.

#### 3.2.2. Impregnation of Cellulose Substrates with PBI@CNTs and/or ZnO Dispersions

**Figure 3:**
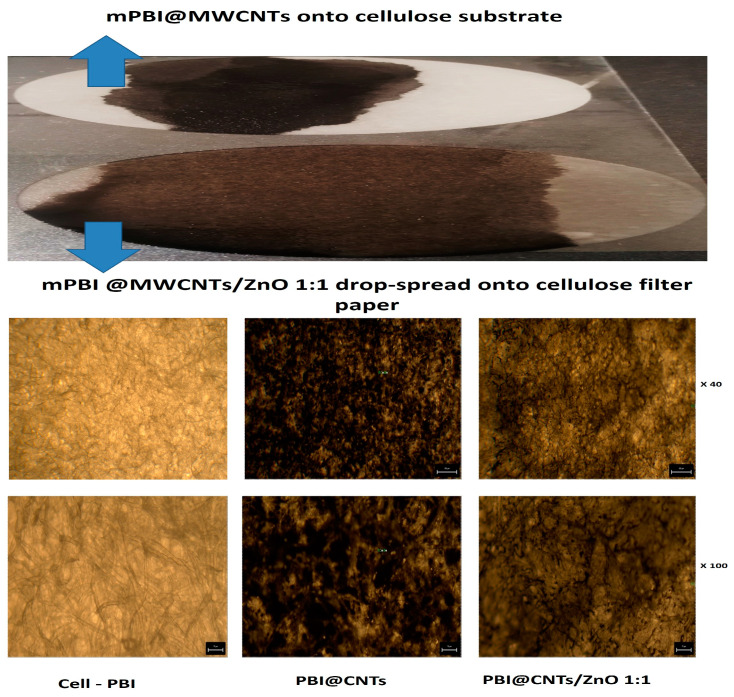
Digital pictures of 1 mL free drop spread of m-PBI@CNT and m-PBI@CNTs/ZnO 1:1 suspensions (**top**) and light microscopy pictures of these two dispersions after 1 mL deposition onto cellulose filter substrate (**bottom**).

Free drop spreading method was used for deposition and impregnation of cellulosic filter paper substrates with both bare PBI@CNTs and with the three different mixed PBI@CNTs/ZnO dispersions (1:1, 1:2, and 1:3 weight ratios) in DMAc. The filter substrate was fixed horizontally onto a glass slide on a heat plate at 80 °C in order to accelerate the organic solvent evaporation and drying/fixation of the carbon nanotube/ZnO layer onto the cellulosic fibers surface. The very prominent positive effect of ZnO particles addition into m-PBI@CNTs dispersion for increased spreading and impregnation efficiency onto the used cellulosic filter substrate was evidenced by the made digital images and by the light microscopy analysis of impregnated substrates (Figure 3). As seen from the pictures, the addition of a 1:1 feed ratio of ZnO particles allows the covering of a higher impregnation area onto the cellulose substrate as compared with pure PBI@CNTs dispersion and with the use of the same amount of deposited impregnating dispersion (1 mL deposition). This observation could be explained by decreased hydrophobicity and increased overall polarity of the mixed CNTs/ZnO dispersions, as well as the purely mechanistic influence of the platelet-shaped ZnO NPs and their dispersion agglomerates onto the anisotropic cylindrical shaped MWCNTs and their hydrophobic and Van der Waals force driven precipitation/fixation onto the abundant polar OH groups anchoring the fibrous cellulosic surface.

#### 3.2.3. XRD Study

**Figure 4:**
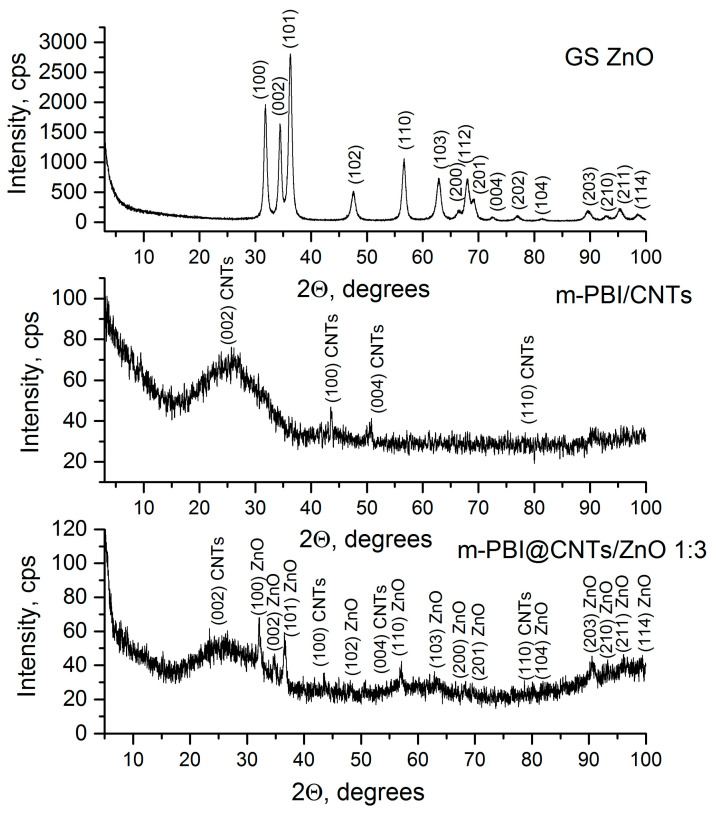
PXRD patterns of green synthesized ZnO, m-PBI/CNTs, and m-PBI@CNTs/ZnO 1:3.

The powder X-ray diffraction patterns of the prepared green synthesized ZnO and m-PBI/CNTs and m-PBI@CNTs/ZnO 1:3 hybrid materials are presented in Figure 4. The existence of the ZnO phase (Ref. Code: 01-074-9940) was registered in pure zinc oxide and m-PBI@CNTs/ZnO. The carbon nanotubes (Ref. Code: 00-058-1638) and PBI amorphous halo are observed for both hybrid materials. The calculated average crystallite size, lattice microstrain parameter, and unit cell parameter of the pristine green synthesized ZnO phase are 15 nm, 2.41 × 10^−3^ a.u., and a: 3.24 Å. 

#### 3.2.4. XPS Study of Impregnated Cellulosic Substrates

**Figure 5:**
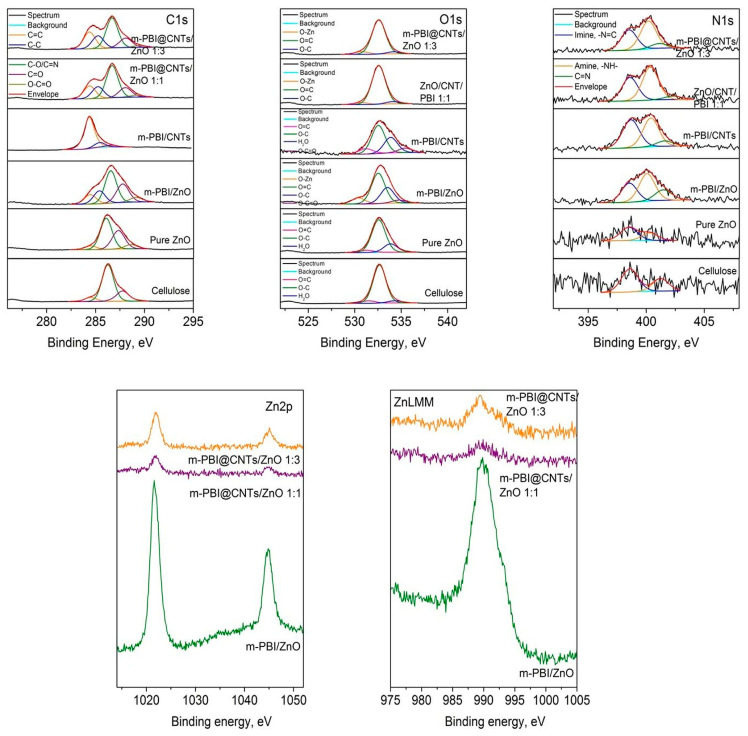
Deconvoluted photoelectron spectra of C1s, O1s, and N1s and core level spectra of Zn2p and ZnLMM of m-PBI/ZnO; m-PBI/CNTs; m-PBI@CNTs/ZnO 1:1; and m-PBI@CNTs/ZnO 1:3.

The XPS results show that the chemical elements C, O, Zn, and N are registered on the surface. The differences in the surface composition (Table 1) and electronic structure of the layers obtained with polymers (PBIs) and carbon nanotubes (CNTs) were investigated. The interpretation of the obtained C, O, and N spectra shows that their shape is asymmetric, which leads to their decomposition with Lorenzian–Gaussian curve fitting. The deconvoluted C1s spectra (Figure 5) show a main peak at ~286.5 eV, which is attributed to C-O/C=N bond [52,53]. An exception is observed in the CNT/PBI sample, where the main peak at 284.4 eV corresponds to sp^2^ carbon in a graphitic structure [54]. The binding energies of the remaining carbon components are presented in Table 2. The position of the maximum of the Zn2p_3/2_ peaks at ~1021.8 eV is associated with the Zn^2+^ oxidation state. The atomic concentrations of C and O were found to be similar, with a difference observed in the concentration of Zn and N. The amount of zinc was greater in the ZnO sample obtained with polymer. This is also proven by the decomposed O1s spectra. The binding energy of the N1s peaks ascribe to amine and imine groups. The amount of nitrogen increased with the addition of CNTs and PBIs, and another peak was observed that was attributed to the organic nitrogen (PBI matrix 399.9 eV ± 1.1). 

#### 3.2.5. SEM and EDS Mapping/Spectra Analysis of the Photocatalyst Impregnated Cellulosic Scaffolds

**Figure 6:**
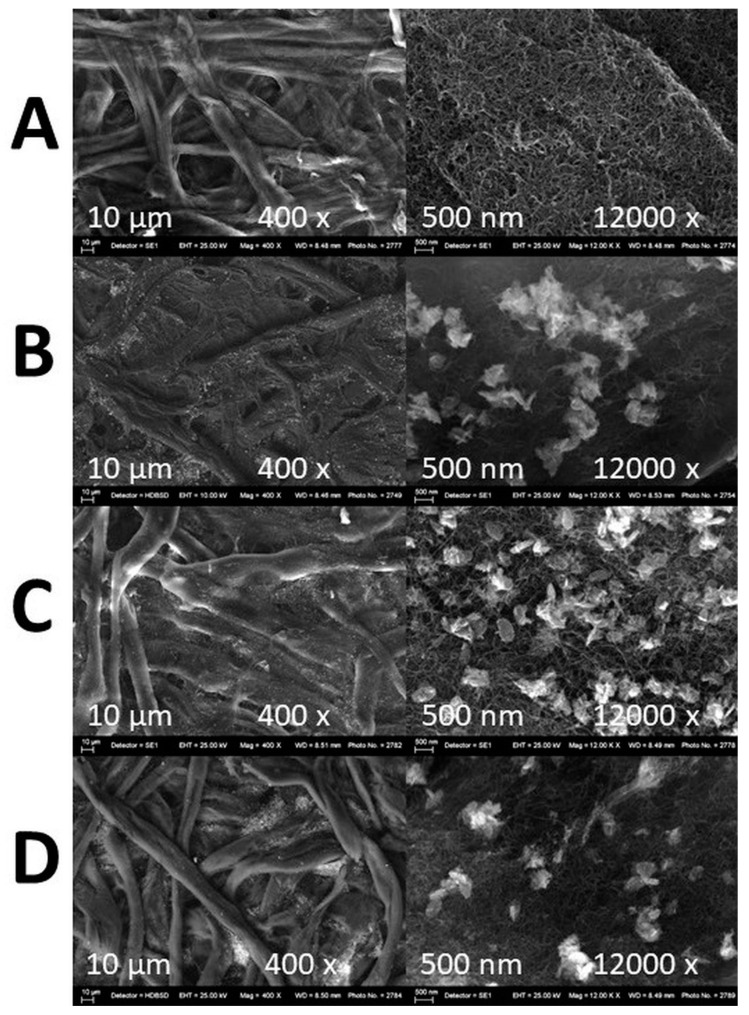
SEM images of microfibrous cellulose substrates impregnated with (**A**) m-PBI@CNTs; (**B**) m-PBI@CNTs/ZnO 1:1; (**C**) m-PBI@CNTs/ZnO 1:2; and (**D**) m-PBI@CNTs/ZnO 1:3.

**Figure 7:**
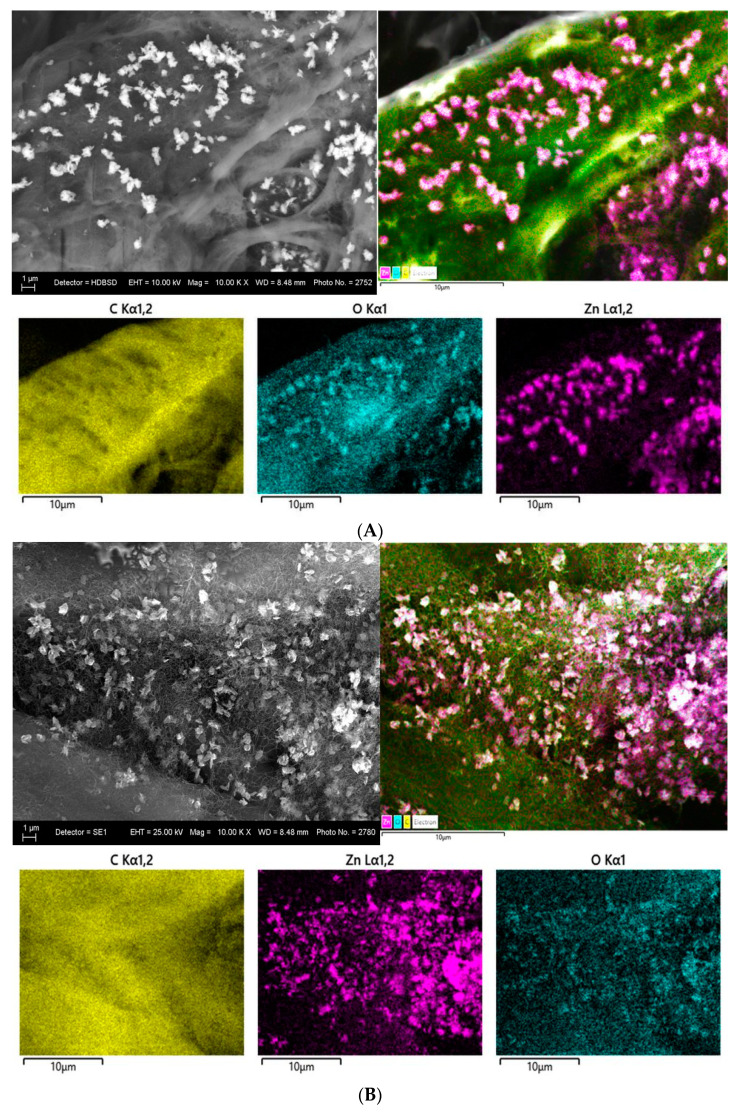

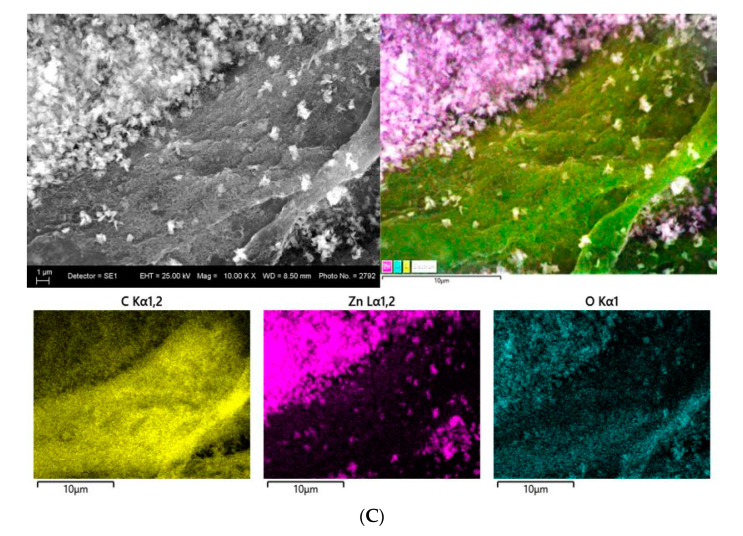
EDS mapping of cellulose substrate impregnated with m-PBI@CNTs/ZnO 1:1 (**A**); m-PBI@CNTs/ZnO 1:2 (**B**); and m-PBI@CNTs/ZnO 1:3 (**C**).

Figure 6 presents the SEM images of the dense cellulosic filtration substrates surface impregnated with the different m-PBI@CNTs dispersions—the pristine one (Figure 6A) and the ZnO NPs containing the three different weight ratios (Figure 6B–D). As could be summarized from the SEM analysis, during the impregnation process, the PBI-wrapped carbon nanotubes are very evenly distributed and infiltrated on the top of the cellulosic substrate, forming a dense cover surrounding the individual cellulosic microfibrils and do not form any observable agglomerated subareas. On the other hand, during the free drop impregnation process, the ZnO nanoplatelets are situated on the top of the CNTs layer with some clustering formation. At the highest 1:3 ZnO NPs feed content, the agglomeration of ZnO NPs is most profound, and at 1:1 and 1:2, the nanoplatelets are more evenly distributed on the top layer of CNTs with small uncovered areas. This could be explained by the explicit hydrophilic–hydrophobic interactions during the filtering impregnation process between the polar micro fibrous cellulosic substrate from one hand and the less-polar CNTs/ZnO nanophases and the relatively low-volatility DMAc solvent itself. The EDS mapping analysis of the prepared m-PBI@CNTs/ZnO impregnated samples is in agreement with the conventional SEM observations as, again, the most clustered areas are observed in the m-PBI@CNTs/ZnO 1:3 impregnated celluloses as seen from Figure 7C images. 

**Figure 8:**
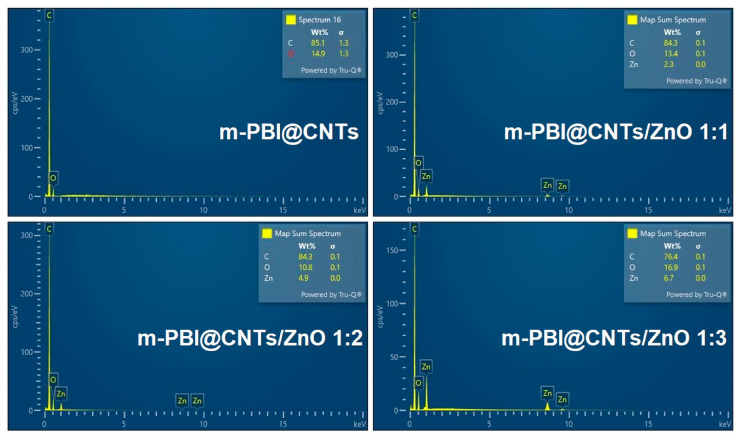
EDS spectra of m-PBI/CNTs, m-PBI@CNTs/ZnO 1:1, PBI@CNTs/ZnO 1:2, and m-PBI@CNTs/ZnO 1:3.

Figure 8 presents EDS spectra of the synthesized m-PBI/CNTs, m-PBI@CNTs/ZnO 1:1, m-PBI@CNTs/ZnO 1:2, and m-PBI@CNTs/ZnO 1:3 materials. The EDS results show the presence of C and O for m-PBI/CNTs and C, O, and Zn peaks for m-PBI@CNTs/ZnO 1:1, m-PBI@CNTs/ZnO 1:2, and m-PBI@CNTs/ZnO 1:3, without other elemental contaminations.

#### 3.2.6. AFM and TEM Analysis

**Figure 9:**
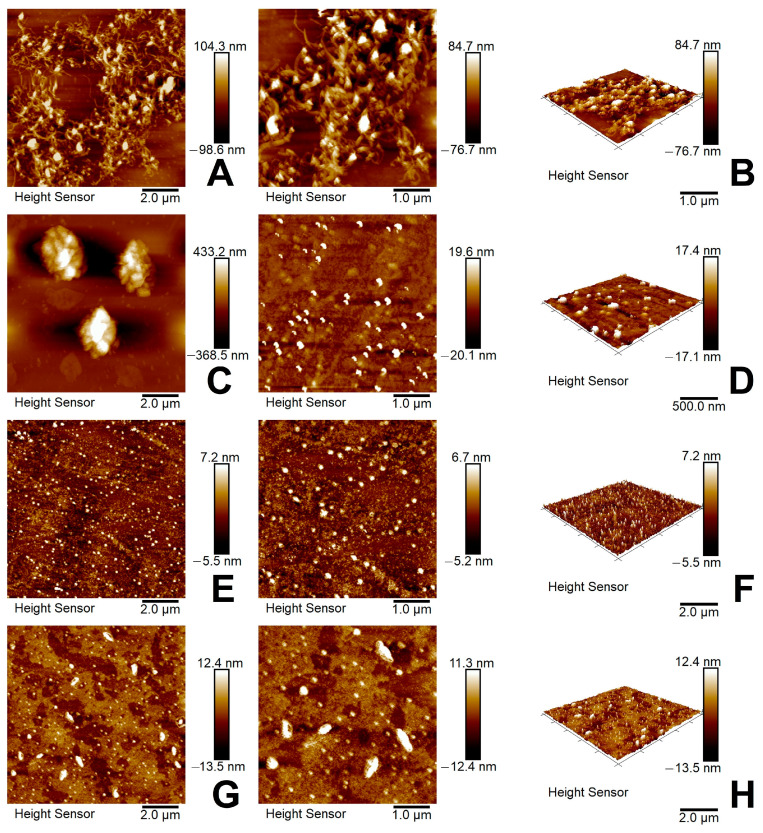
(**A**) AFM 2D image of CNTs; (**B**) AFM 3D image of CNTs; (**C**) AFM 2D image of green synthesized ZnO nanoparticles; (**D**) AFM 3D image of green synthesized ZnO nanoparticles; (**E**) AFM 2D image of m-PBI/ZnO; (**F**) AFM 3D image of m-PBI/ZnO; (**G**) AFM 2D image of m-PBI@CNTs/ZnO; (**H**) AFM 3D image of m-CNTs/ZnO.

The surface topography images of carbon nanotubes, ZnO nanoparticles, m-PBI/ZnO, and CNTs/ZnO samples evaluated by AFM are shown in Figure 9. The randomly distributed large objects with fibrillar morphology typical for the matrix of carbon nanotubes are shown in Figure 9A,B. The morphology of green synthesized ZnO layers represents regularly distributed domains of ZnO nanoparticles of size 0.1–0.2 µm and barely situated domains of ZnO particles of size 1.0–2.0 µm (Figure 9C,D). Consequently, the films of hybrid m-PBI/ZnO samples show smooth and uniform surfaces of coated material containing even ZnO domains of size 0.1–0.2 µm (Figure 9E,F). Oppositely, the films of hybrid CNTs/ZnO samples present a more rough and non-uniform surface of coated material enclosing larger CNTs/ZnO domains of size 0.2–0.5 µm (Figure 9G,H).

**Figure 10:**
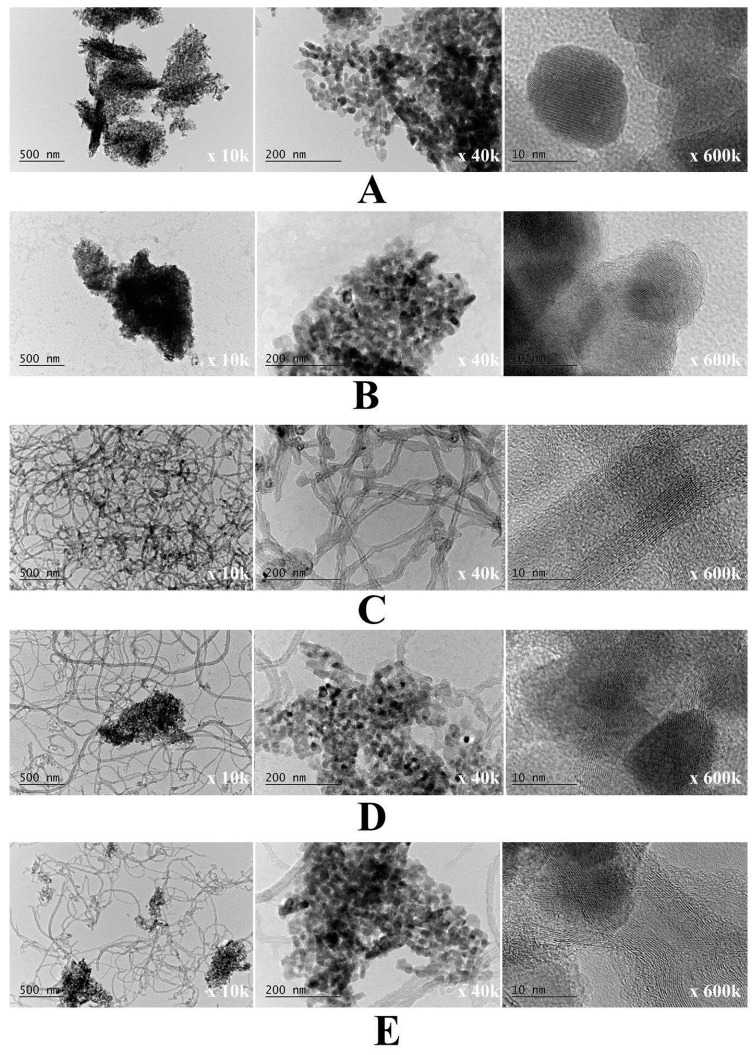
TEM images of (**A**) green synthesized ZnO nanoparticles; (**B**) m-PBI/ZnO; (**C**) m-PBI/CNTs; (**D**) m-PBI@CNTs/ZnO 1:1; and (**E**) m-PBI@CNTs/ZnO 1:3.

**Figure 11:**
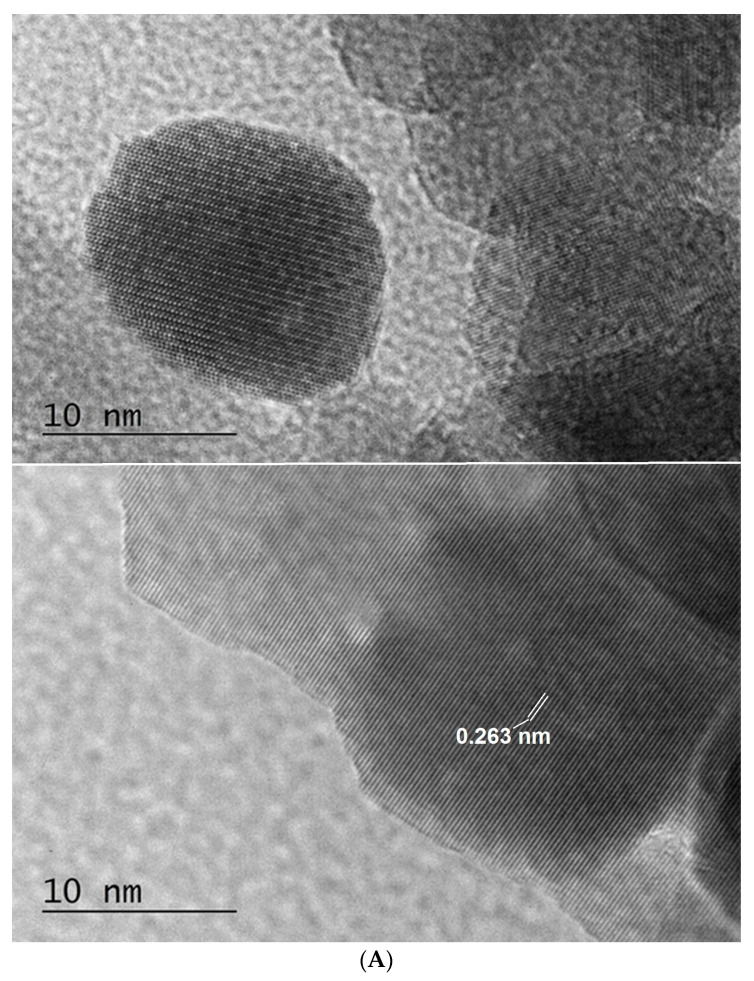

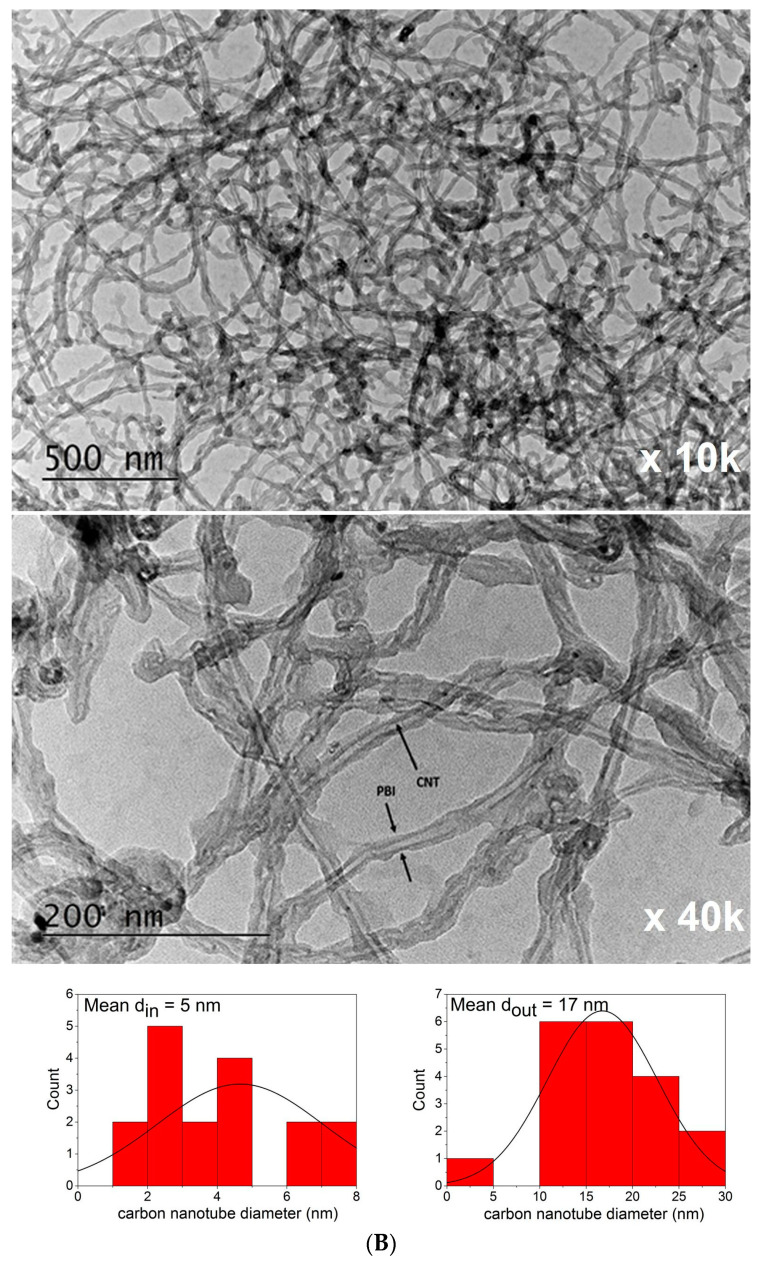
HR TEM images of images of green synthesized ZnO nanoparticles (**A**). m-PBI@CNTs hybrid (**B**).

The fine structure and morphology of the bare ZnO and m-PBI@ZnO, as well as the hybrid m-PBI@CNTs and PBI@CNTs/ZnO native dispersions, were studied by TEM analysis as shown in Figure 10. In this case, in opposite to AFM analysis sample preparation, where almost immediate solvent evaporation of deposition dispersions during spin coating occurs, for TEM analysis, we used standard TEM grids dip and slow solvent evaporation technique, which could lead to the occurrence of some additional drying agglomeration phenomena of both CNTs and the ZnO phase though the performed DLS analysis already showed indication for the inherent existence such agglomerated phase, especially for the ZnO containing dispersions. At a certain point, during the slow evaporation of DMAc solvent from the PBI-stabilized bare ZnO dispersion, the resulting concentration of the PBIs in the presence of ZnO NPs seems to lead to the occurrence of threshold ionotropic gelation of the composite PBI matrix and the formation of relatively big agglomerates of nanoparticles comparable by size with those of the bare ZnO agglomerates (Figure 10A,B). With increasing the ZnO content in the mixed m-PBI@CNTs/ZnO dispersions, the occurrence of higher agglomerates is obvious in Figure 10D,E. In Figure 11A, the high-resolution TEM images of green synthesized ZnO NPs prepared by plant extract hydrothermal methodology, one can clearly observe the crystal planes of ZnO. The lattice plane fringes of the ZnO nanoparticles are used to calculate the d-spacing values, which in our case is about 0.263 nm, indicating the formation of ZnO nanocrystals. The interplanar spacing of the green synthesized ZnO NPs corresponds well to the (002) planes of wurtzite ZnO [55]. The HR TEM images of the PBI surface-stabilized MWCNTs in Figure 11B showed a high degree of debundling and interlaying of individual CNTs with avr. core diameter of 5 nm and avr. outer diameter of 17 nm. The formation of a diffusive PBI-wrapped layer onto the surface of the CNTs is also well visible. 

### 3.3. Photocatalytic Study of Prepared PBI-Stabilized Carbon Nanotubes/GREEN Synthesized ZnO Composites 

**Figure 12:**
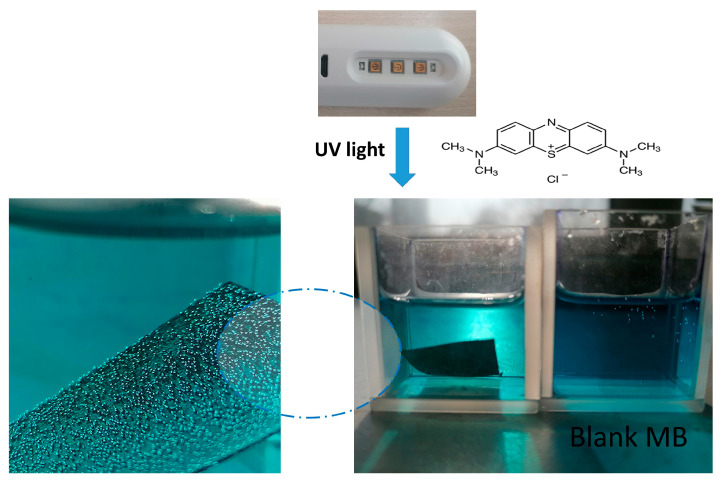
Digital pictures of preliminary experiment for 15 min UV light illumination of hybrid catalytic m-PBI@CNT/ZnO 1:1 cellulose substrate in contact with diluted MB solution.

Our preliminary test of the prepared hybrid catalyst m-PBI@CNT/ZnO 1:1 cellulose substrate put in short contact with methylene blue solution and illuminated with low power diode UV bactericidal lamp (3 W) showed continuous gas bubble formation from the hybrid catalyst surface and decrease in color intensity of the dye solution (Figure 12). This was an obvious indication of the generation of reactive oxygen species (ROS) from the impregnated catalyst substrate. In the case of one component, m-PBI@CNTs impregnated cellulose, the bubble formation was less profound, which was an indication of the presence of synergism between m-PBI@CNTs and the plant extract ZnO NPs. 

**Figure 13:**
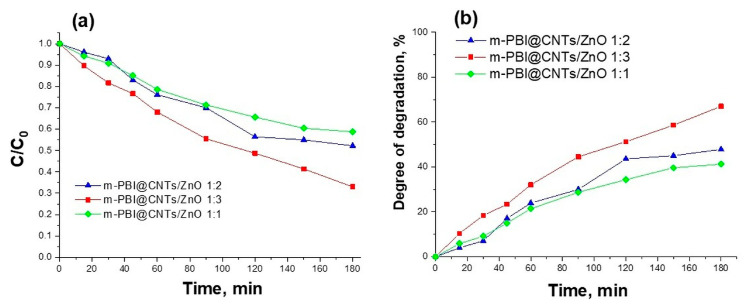
(**a**) The concentration ratio C/C_0_ and (**b**) degree of degradation of Methylene Blue dye with time of UV illumination using prepared hybrid materials as photocatalysts.

The photocatalytic degradation of methylene blue dye using the as-synthesized m-PBI@CNTs/ZnO hybrid materials. Figure 13 presents the concentration changes C/C_0_ and the degree of degradation of methylene blue dye as a function of the time of UV irradiation. 

**Figure 14:**
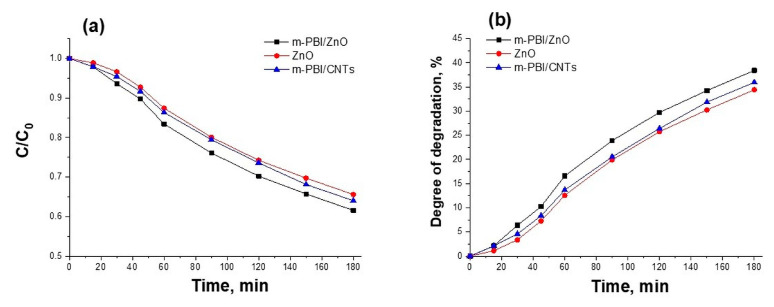
(**a**) The concentration ratio C/C_0_ and (**b**) degree of degradation of Methylene Blue dye with time of UV illumination using m-PBI/ZnO and bare green synthesized ZnO as photocatalysts.

The photocatalytic tests have determined that in the course of the photocatalytic reaction, the m-PBI@CNTs/ZnO 1:3 photocatalyst leads to a higher degree of degradation of the methylene blue dye (67%) in comparison with the other two investigated m-PBI@CNTs/ZnO 1:2 and 1:1 composites (48% and 41%). The photocatalytic activity of m-PBI@CNTs/ZnO hybrid materials is enhanced with increasing ZnO content. The pure ZnO and m-PBI/ZnO demonstrate a lower photocatalytic ability than that of synthesized meta-PBI-stabilized carbon nanotubes/zinc oxide hybrid materials (Figure 14). The pristine m-PBI and cellulose substrate show no photocatalytic activity toward the degradation of methylene blue dye under UV illumination. 

The photocatalytic activity of m-PBI@CNTs/ZnO hybrid materials is favorably affected by several factors: (i) MWCNTS in hybrid photocatalyst acts as a dispersive of nanoparticles and prevents zinc oxide nanoparticles from additional hunching and agglomeration. As a result, the active surface of ZnO (NPs)/MWCNTs can be increased [56]; (ii) MWCNTs act as an electron acceptor from ZnO, significantly hindering the recombination of charge carrier and suppression of photocorrosion of ZnO thus improving photocatalytic activity [57]; (iii) The presence of PBIs, as well as the occurrence of interfacial charge transfer and separation between the conjugated polymer (PBI) and the conductor/semiconductor (MWCNTs/ZnO) ensures that the photo responsiveness to UV light is improved [58]; (iv) it is inferred that the combination or mutual hybridization of cellulose with different photocatalysts not only alters structural properties but also enhances their photocatalytic ability by providing more surface area for the adsorption of pollutant target molecules [59]. Despite the relatively very low concentration of the cellulose deposited photocatalyst (in the range 0.023 mg/cm^2^ for the bare PBI@CNTs and 0.023–0.069 mg/cm^2^ for the mixed PBI@CNTs/ZnO) and the low power of the UV source (18 W), the investigated composites demonstrate relatively high photocatalytic activity (41–67%). In comparison, in a similar photocatalyst material study, a larger amount of 50 mg MWCNT/ZnO:Al:N photocatalyst coated on 1 cm^2^ glass substrate sample and 100 W Hg lamp irradiation resulted in a similar degree of degradation of MB dye of about 65% achieved [60]. 

According to the literature data [29], the degradation mechanism of Methylene Blue presents that irradiation of the nanocomposite with UV light transfers the occupied valence band (VB) electrons of the ZnO to a conduction band (CB). The CB contains electrons (e^−^), and the VB holds the holes (h^+^). The hole–electron pairs are produced on the surface of the ZnO. The surface electrons quickly move to the edge of the MWCNTs, and the holes remain in the ZnO. Water molecules combine with VB holes to generate hydroxyl radicals, and dissolved oxygen molecules (from water) produce superoxide radicals with the assistance of photogenerated electrons in the CB. MWCNTs slow the recombination efficiency between holes and electrons and accelerate the redox reaction. In addition, ^•^$O_{2}^{-}$ free radicals generate ^•^OH free radicals. Furthermore, the VB-containing holes can contact the ^−^ OH groups adsorbed onto the surface to generate ^•^OH free radicals. The degradation of MB dye can be followed by the direct interaction between holes and MB. As a result, strong oxidants such as ^•^OH free radicals degrade dye [29].

**Figure 15:**
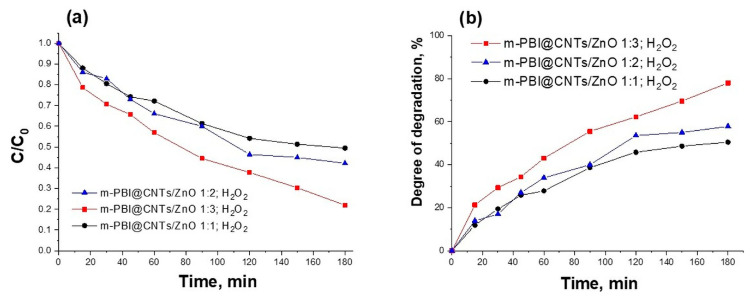
(**a**) The concentration ratio C/C_0_ and (**b**) degree of degradation of Methylene Blue dye with time of UV illumination using prepared hybrid materials as photocatalysts in the presence of H_2_O_2_.

The addition of H_2_O_2_ during the photocatalytic experiments leads to increasing the photocatalytic ability of all tested m-PBI@CNTs/ZnO hybrid photocatalysts, as shown in (Figure 15). The degree of degradation of MB dye in the presence of H_2_O_2_ increases (50–78%) compared to the absence of H_2_O_2_ (41–67%). This can be explained by the fact that hydrogen peroxide acts as a source of hydroxyl radicals and as an electron scavenger, thus inhibiting electron–hole recombination. In addition, H_2_O_2_ acts as an alternative electron acceptor to oxygen since H_2_O_2_ is a better electron acceptor than molecular oxygen [61].

**Figure 16:**
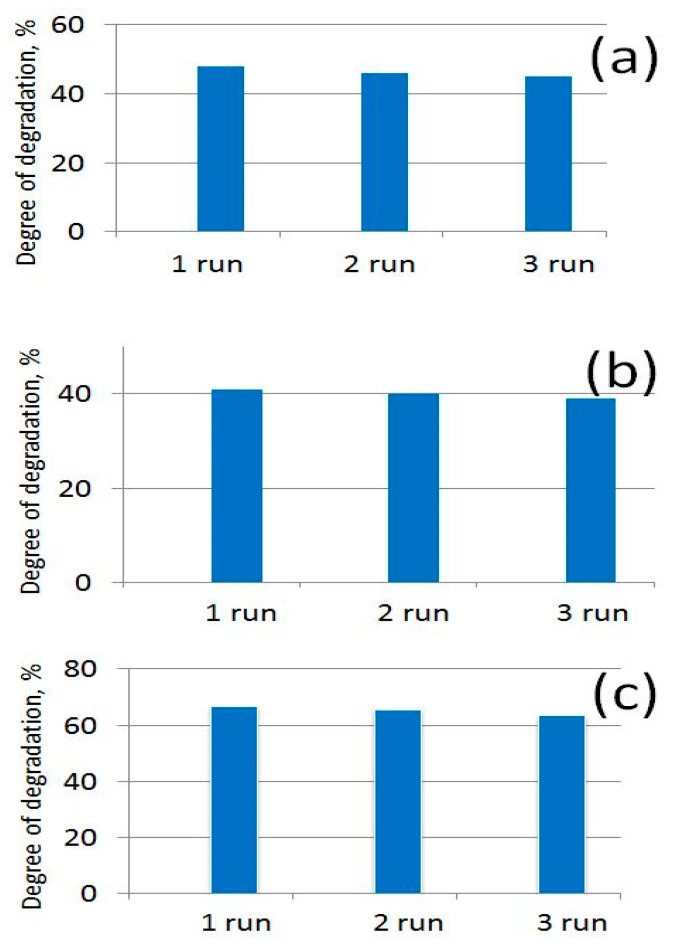
Degree of degradation of MB dye after 180 min under UV light using (**a**) m-PBI@CNTs/ZnO 1:2; (**b**) m-PBI@CNTs/ZnO 1:1; and (**c**) m-PBI@CNTs/ZnO 1:3 photocatalysts in three photocatalytic runs.

It has also been established that the tested photocatalysts retained their photocatalytic activity relatively well after three photocatalytic cycles, which show their suitable long-term stability (Figure 16).

### 3.4. Antibacterial Activity of Prepared PBI-Stabilized Carbon Nanotubes/Green Synthesized ZnO Composites 

Before testing the antibacterial properties of the bare MWCNTs and plant extract ZnO nanopowders, as well as the hybrid cellulosic scaffold materials, they were sterilized and tested for the release of free solutes with their own antimicrobial activity. In this control experiment, no sterile zones were formed after 24 h of cultivation. This result indicated that neither the hybrid materials nor their constituents release a considerable amount of soluble substances with an antibacterial effect, at least not in the threshold concentration needed to cause such an effect. In a similar experiment with ZnO nanoparticles, ref. [62] obtained the same results against several test microorganisms, including *E. coli*, while with Staphylococcus aureus they observed a sterile zone. Apparently, this bacterium is sensitive to small amounts of Zn^2+^ that pass into the agar layer.

Most antimicrobial agents widely used to date target a specific mechanism in the bacterial cell—for example, cell wall synthesis, protein or nucleic acid synthesis, change in cell membrane permeability, etc. This enables the bacterial cell to adapt by using various resistance mechanisms, for example, the synthesis of enzymes that degrade the antimicrobial agent, changes in the structure of the cell wall, or in the transport systems of the cell membrane [35], i.e., specific impact on a well-defined target results in a specific response from the bacteria. The increasing drug resistance of pathogenic microorganisms is one of the most alarming modern challenges in the field of medicine, and the creation of polymers with highly effective antimicrobial properties is increasingly emerging as one of the solutions to this problem [63]. The mechanism of action of antimicrobial ingredients in composite materials is not specific, i.e., it does not affect specific biochemical pathways in the living cell but leads to the indiscriminate destruction of macromolecules from virtually all cellular structures. In addition, in the course of their action, they do not undergo chemical changes that lead to their neutralization. These two factors contribute to the broad spectrum of action of the active ingredients of the composites and prevent microorganisms from developing resistance to them.

**Figure 17:**
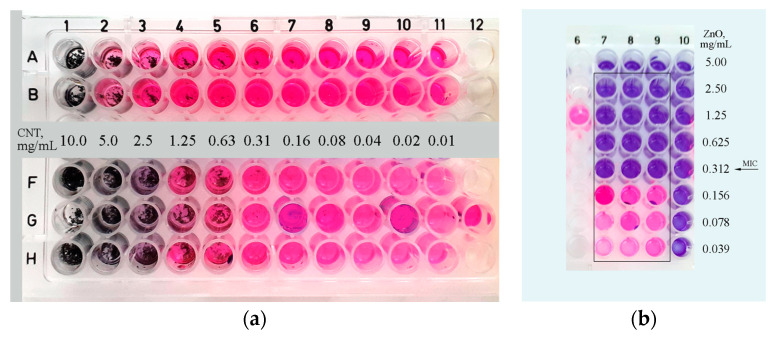
RMDA method for evaluation of MIC in 96-well plate. Wells in the rectangles are inoculated with a bacterial culture of 5 × 10^5^ CFU/mL. (**a**) MIC of MWCNTs, lines A, B: a sector from 96-well plate kept in static condition. Lines F–H: a sector from another 96-well plate incubated on a wrist shaker. Legend between B and F lines shows the concentration of MWCNTs in each column of wells. Well G12—positive control. (**b**) MIC of ZnO nanoparticles, column 6: positive control, column 10: control wells of resazurin dye for each ZnO concentration.

The performed quantitative RMDA method showed that the carbon nanotube powder has no antimicrobial activity toward the *E. coli* strain at the loaded concentration of 10 mg/mL. The highly agglomerated and hydrophobic powder settles to the bottom of the well and apparently does not make effective surface contact with the bacterial cells, which explains the lack of any substantial antibacterial effect. In order to check this hypothesis suggestion, we performed a slight modification of the method, incubating the RMDA plate demand on a wrist-action shaker. A profound antimicrobial activity of MWCNTs at a concentration of 5 mg/mL was observed in this case (Figure 17a). We hypothesize that the constant movement increases the probability of contact between the nanotubes’ surface or with their sharp end tips and the bacterial cells, leading to mechanical damage to their cell wall. A similar phenomenon is already observed by [64].

Using the same method, we found that zinc oxide nanoparticles’ MIC for *E. coli* is 0.312 mg/mL and 0.06 mg/mL for *B. subtilis*. Compared to the results of other authors obtained with the same method (MICs between 6 and 50 µg/mL, refs. [62,65]), the value for *E. coli* is significantly higher. Zinc oxide nanoparticles also affect cells by direct contact, but unlike carbon nanotubes, there is apparently no explicit need for this. 

**Figure 18:**
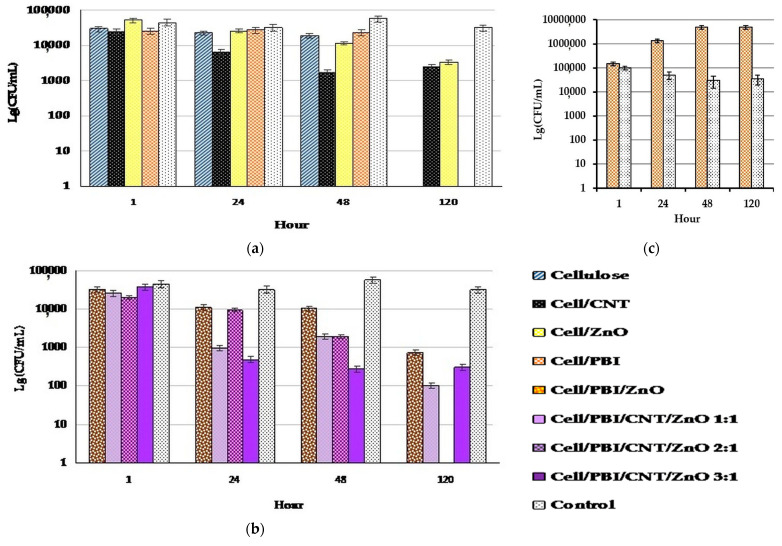
ASTM Standard Test Method E 2149–10. Data are presented as lg of the CFU/mL. (**a**,**b**) Antibacterial effect of the tested hybrid materials, their constituents, and combinations of constituents tested on *E. coli*. (**c**) Effect of the combination of cellulose and PBIs, tested on *B. subtilis*.

The standard ASTM test method enables the dynamic contact between the surface of the material and the bacterial cells in an aqueous environment. Through this method, we observed the specific effect of each sample on the test bacterium *E. coli*, followed over a period of 120 h. The data are presented in Figure 18. As expected, the cellulose membrane alone did not exhibit antibacterial activity. Nanotubes spread over the cellulose sheet have a well-expressed antimicrobial effect—the amount of living cells decreases by about 50% at the 48th hour, which indicates that the fixation of these nanoparticles to a carrier enables a “hard” contact between them and the cells. As first described by [64], cells floating freely in the liquid were not damaged by the nanotubes, while those in direct contact with them suffered mechanical cell wall damage and died in over 80% [64].

The combination of cellulose and zinc oxide nanoparticles shows interesting results of antimicrobial activity, which manifests itself later and more smoothly increases over time. Furthermore, it could not reach the effect of pure ZnO NPs even after 120 h. Some of them probably fall deep between the cellulose fibers, and their direct contact with the cells is prevented. In addition, it is possible that some of the ROS act on the cellulose fibers and are thus neutralized by oxidizing them.

The combination of cellulose and PBIs had no antimicrobial activity. It should be noted that here, the number of CFU remains unchanged until the 48^th^ hour. The absence of live cells at 120 h may be due to natural cell death for other reasons, as CFUs are absent at this hour in some samples that show antimicrobial activity during the rest of the experiment and in some that do not (cellulose, cellulose–PBI, cellulose–PBI/ZnO:CNT 2:1). The constant number of CFU observed with this combination led us to conduct a separate experiment only for its effect on *Bacillus subtilis*, although the standard method requires that the antimicrobial activity be tested on *E. coli*. An even more interesting result was obtained with this bacterium—the number of CFU increases over time (Figure 18c). Therefore, we could say that PBI either has no effect or has a beneficial effect on bacterial cells. To the best of our knowledge, such an experiment with PBI was conducted for the first time, and the data obtained from it are new. Further experiments are needed to investigate this effect. 

All three combined materials with an increasing ratio of zinc oxide to CNT showed higher antimicrobial activity than the combination without nanotubes. The test replicates in this experiment did not allow us to observe an increase in the effect with increasing amounts of zinc oxide. This is probably due to local unevenness in the distribution of zinc oxide NPs on the small-area samples used in the tests.

#### Photocatalytic Antibacterial Activity

**Figure 19:**
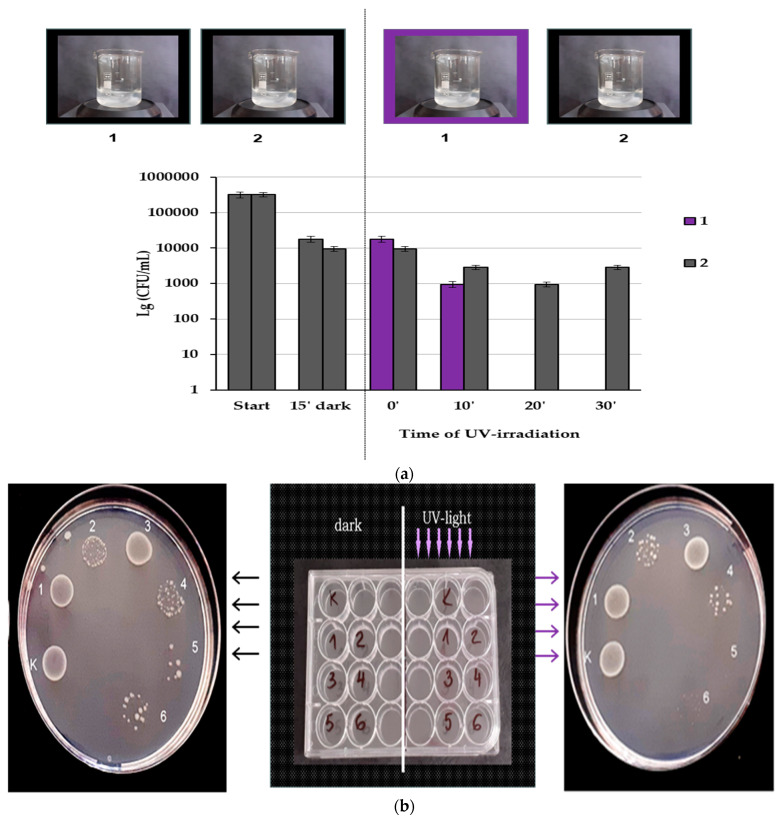
(**a**). Effect of UV irradiation on standard *E. coli* suspension with 0.5 mg/mL ZnO NPs. Samples were taken before the 15′ incubation in the dark, at the start of UV irradiation, and at 10′, 20′, and 30′: (1) a beaker irradiated with UV light; (2) a beaker kept in the dark throughout the whole experiment; (**b**) effect of the tested hybrid materials: left—without irradiation; right—after UV irradiation; K—control, 1—Cell/PBI, 2—Cell/PBI/ZnO, 3—Cell/PBI@CNT, 4—Cell/PBI@CNT/ZnO 1:1, 5—Cell/PBI@CNT/ZnO 1:2, 6—Cell/PBI@CNT/ZnO 1:3. Sample 5 displays bactericidal effect.

Zinc oxide nanoparticles exhibit photocatalytic activity, which is expressed in the much more active production of ROS under UV irradiation than in the dark. To distinguish the effects of these ZnO NPs with and without UV irradiation, we performed an experiment in which they were placed in constant motion for more effective cell contact and to prevent sedimentation while simultaneously testing the effect of a UV-irradiated sample and one placed in the dark. The photocatalytic effect was tracked and achieved in a short time—from 0 to 30 min, and after 20 min, no CFUs were registered (Figure 19a). In contrast, without UV light, only bacteriostatic effect could be observed for the time interval used.

The photocatalytic activity of the hybrid materials was tested after static incubation of a standard suspension of *E. coli* in wells containing 0.5 cm^2^ of the materials in a 24-well plate. After incubation of the plate overnight at 30 °C and irradiation for 10 min, bacterial growth on a solid medium was observed. The results are shown in Figure 19a,b. Apparently, all materials containing zinc oxide show antibacterial activity even without irradiation (Figure 19b), reflecting the intrinsic action of zinc oxide. Figure 19b shows that irradiation produces an additional antibacterial effect of the same materials, which reaches growth inhibition (Cell/PBI/CNT:ZnO 1:3) or complete destruction of bacteria (Cell/PBI/CNT:ZnO 1:2).

**Figure 20:**
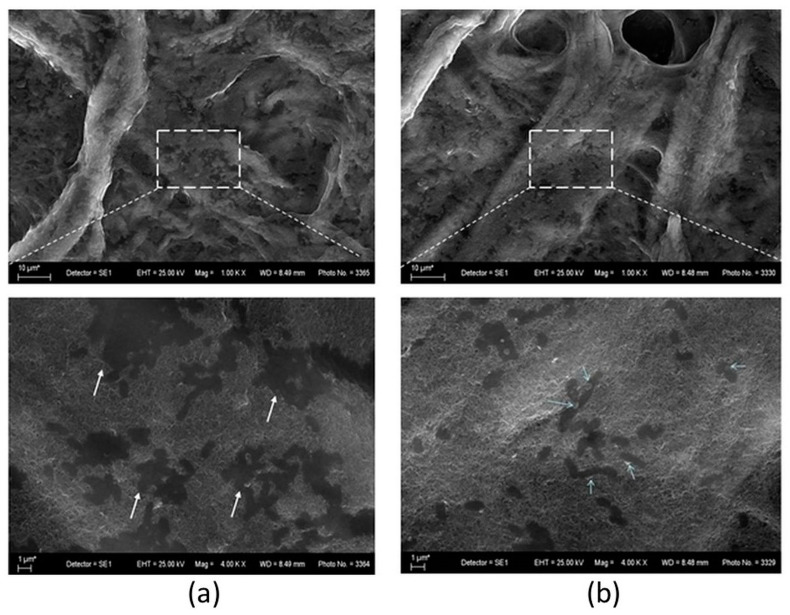
SEM images of the hybrid material Cell/PBI@CNT incubated with *E. coli* suspension overnight. Putative effusions of cell contents are indicated by arrows. Areas marked in rectangles were observed at higher magnification and shown below: (**a**) a specimen kept in the dark; (**b**) a specimen irradiated by UV light.

The combination of PBIs and MWCNTs on a cellulose carrier proved suitable for the observation of the test microorganisms before and after UV light irradiation using SEM (Figure 20). Comparison with photographs of similar hybrid material in the literature [66] gave us reason to assume that the images obtained by us show cells of the typical *E. coli* shape and size, but their appearance is more like footprints or cell shadows. At higher magnification, some of the cells showed granules of a different material that resembled an effusion of cell contents. Observation at lower magnification makes it possible to observe that these fine granules, appearing brighter than the rest of the objects, are observed in greater abundance at the specimen irradiated with ultraviolet light than at the one kept in the dark. Three-dimensional cylindrical rod-shaped cells are not observed, as undamaged cells should appear. These features lead us to assume that the cells on the surface of the nanotubes are completely destroyed as a result of their direct contact with the nanoparticles, consistent with the results of [64]. This was probably due to mechanical damage to the cell wall, which led to cell rupture and leakage of cytoplasmic matter. Additional damage that produced the same result was apparently caused by exposure to ultraviolet light. 

## 4. Conclusions

Novel hybrid materials based on m-PBI-stabilized multi-walled CNTs and mixed CNTs/green synthesized ZnO NPs dispersions were prepared and used for surface impregnation of microfibrous cellulose substrate. The addition of ZnO NPs greatly improves the impregnation/spreading efficiency of MWCNT dispersion onto cellulosic substrate. The mean crystallite size of prepared green synthesized ZnO determined by XRD analysis is 15 nm. Agglomerates from clustered individual ZnO NPs with sizes in the range of 250–350 nm were observed for m-PBI@CNTs/ZnO dispersions deposited onto cellulosic fibers. The obtained hybrid CNTs/ZnO cellulosic substrates showed photocatalyst activity toward MB dye UV degradation despite the relatively low catalyst loading. The highest degree of degradation of the methylene blue dye (67%) was achieved using m-PBI@CNTs/ZnO 1:3 photocatalyst. The degree of degradation of MB dye in the presence of diluted H_2_O_2_ increases (50–78%) compared to experiments without H_2_O_2_ addition (41–67%). The combination of biologically inert cellulosic filter paper, PBI as a stabilizing agent, and the two types of nanoparticles showed significant antibacterial activity, as well as synergism of PBI@CNTs and the ZnO NPs. The hybrid scaffolds’ ability to induce the release of ROS is increased many times upon irradiation with UV light and greatly reduces the time required to achieve a significant bactericidal effect. 

## Figures and Tables

**Table 1:** XPS results of quantitative composition of m-PBI@CNTs/ZnO.

Samples	C, at.%	O, at.%	N, at.%	Zn, at.%
Cellulose	57.3	42.3	0.4	-
Pure ZnO	57.5	42.0	0.4	0.1
m-PBI/CNTs	59.9	33.1	3.5	3.5
m-PBI/ZnO	89.8	4.6	5.6	-
m-PBI@CNTs/ZnO 1:1	65.1	31.6	3.0	0.3
m-PBI@CNTs/ZnO 1:3	66.2	29.4	3.9	0.5

**Table 2:** The percentages of C from various species.

Binding Energy, eV	Chemical Bonding	Concentration, %		
	Cellulose and Pure ZnO	Cellulose	Pure ZnO	
284.5	C=C	7.9	6.2	
286.2	C-O/C=N	70.2	52.7	
287.5	C=O	21.9	33.5	
288.6	O-C=O	-	7.6	
	ZnO-CNT-PBI	ZnO-CNT-PBI 3:1	ZnO-CNT-PBI 1:1	ZnO-PBI
284.4	C=C sp^2^	23.4	18.6	11.9
285.3	C-C sp^3^	17.7	17.1	16.3
286.7	C-O/C=N	42.0	46.6	40.7
287.8	C=O	14.1	14.9	24.9
289.2	O-C=O	2.8	2.7	6.21
	CNT-PBI	CNT-PBI		
284.4	C=C sp^2^	81.9		
285.3	C-C sp^3^	12.6		
286.7	C-O/C=N	5.5		

## Data Availability

Data are included in the manuscript.

## Supplementary Materials

The following supporting information can be downloaded at https://www.mdpi.com/article/10.3390/nano14161346/s1, Video S1: Video cap PBI MWCNTs in DMAc Brownian motion.

## References

[1] Mary Rajaitha P., Shamsa K., Sheebha I., Vidhya B., Maheskumar V., Rajesh S. (2019). Influence of the Positioning of the Incorporated Carbon Nanostructures on the Morphology and Photocatalytic Activity of Microwave Synthesized ZnO Nanorods. J. Nanosci. Nanotechnol..

[2] Malekkiani M., Jannat Magham A.H., Ravari F., Dadmehr M. (2022). Facile fabrication of ternary MWCNTs/ZnO/Chitosan nanocomposite for enhanced photocatalytic degradation of methylene blue and antibacterial activity. Sci. Rep..

[3] Dindar B., Içli S. (2001). Unusual photoreactivity of zinc oxide irradiated by concentrated sunlight. J. Photochem. Photobiol. A Chem..

[4] Shinde D., Tambade P., Chaskar M., Gadave K. (2017). Photocatalytic degradation of dyes in water by analytical reagent grades ZnO, TiO_2_ and SnO_2_: A comparative study. Drink. Water Eng. Sci. Discuss..

[5] Mohd Adnan M., Muhd Julkapli N., Amir M., Maamor A. (2018). Effect on different TiO_2_ photocatalyst supports on photodecolorization of synthetic dyes: A review. Int. J. Environ. Sci. Technol..

[6] Han C., Yang M., Weng B., Xu Y. (2014). Improving the photocatalytic activity and antiphotocorrosion of semiconductor ZnO by coupling with versatile carbon. Phys. Chem. Chem. Phys..

[7] Kusiak-Nejman E., Wojnarowicz J., Morawski A.W., Narkiewicz U., Sobczak K., Gierlotka S., Lojkowski W. (2021). Size-dependent effects of ZnO nanoparticles on the photocatalytic degradation of phenol in a water solution. Appl. Surf. Sci..

[8] Krishnamoorthy A., Varghese S. (2015). Role of surfactants on the stability of nano-zinc oxide dispersions. Particul. Sci. Technol..

[9] Hosseini Largani S., Akbarzadeh Pasha M. (2017). The effect of concentration ratio and type of functional group on synthesis of CNT–ZnO hybrid nanomaterial by an in situ sol–gel process. Int. Nano Lett..

[10] Sui J., Li J., Li Z., Cai W. (2012). Synthesis and characterization of one-dimensional magnetic photocatalytic CNTs/Fe_3_O_4_–ZnO nanohybrids. Mater. Chem. Phys..

[11] Zhang J., Dai M., Zhang S., Dai M., Zhang P., Wang S., He Z. (2022). Recent Progress on Carbon-Nanotube-Based Materials for Photocatalytic Applications: A Review. RRL Sol..

[12] Byrne M.T., Gun’ko Y.K. (2010). Recent Advances in Research on Carbon Nanotube–Polymer Composites. Adv. Mater..

[13] Chen C.S., Liu T.G., Lin L.W., Xie X.D., Chen X.H., Liu Q.C., Liang B., Yu W.W., Qiu C.Y. (2013). Multi-walled carbon nanotube-supported metal-doped ZnO nanoparticles and their photocatalytic property. J. Nanopart. Res..

[14] Araújo E.S., Pereira M.F.G., da Silva G.M.G., Tavares G.F., Oliveira C.Y.B., Faia P.M. (2023). A Review on the Use of Metal Oxide-Based Nanocomposites for the Remediation of Organics-Contaminated Water via Photocatalysis: Fundamentals, Bibliometric Study and Recent Advances. Toxics.

[15] Mallakpour S., Khadem E. (2016). Carbon nanotube–metal oxide nanocomposites: Fabrication, properties and applications. Chem. Eng. J..

[16] Madhusudhana Reddy M., Ramanjaneya Reddy G., Chennakesavulu K., Sundaravadive E., Prasath S.S., Rabel A.M., Sreeramulu J. (2017). Synthesis of zinc oxide and carbon nanotube composites by CVD method: Photocatalytic studies. J. Porous Mater..

[17] Powers E.J., Serad G.A., Seymour R.B., Kirshenbaum G.S. (1986). History and development of polybenzimidazoles. High Performance Polymers: Their Origin and Development.

[18] Kausar A. (2019). Polybenzimidazole-based nanocomposite: Current status and emerging developments. Polym. Plast. Technol. Mater..

[19] Wu J.-F., Lo C.-F., Li L.-Y., Li H.-Y., Chang C.-M., Liao K.-S., Hu C.-C., Liu Y.-L., Lue S.J. (2014). Thermally stable polybenzimidazole/carbon nano-tube composites for alkaline direct methanol fuel cell applications. J. Power Sources.

[20] Sudhaik A., Raizada P., Ahamad T., Alshehri S.M., Nguyen V.-H., Van Le Q., Thakur S., Kumar Thakur V., Selvasembian R., Singh P. (2023). Recent advances in cellulose supported photocatalysis for pollutant mitigation: A review. Int. J. Biol. Macromol..

[21] Gupta K., Sharma B., Garg V., Neelratan P.P., Kumar V., Kumar D., Sharma S.K. (2024). Enhanced photocatalytic degradation of methylene blue and methyl orange using biogenic ZnO NPs synthesized via *Vachellia nilotica* (Babool) leaves extract. Hybrid Adv..

[22] Olaitan Ogunyemi S., Abdallah Y., Zhang M., Fouad H., Hong X., Ibrahim E., Islam Masum M.M., Hossain A., Mo J., Li B. (2019). Green synthesis of zinc oxide nanoparticles using different plant extracts and their antibacterial activity against *Xanthomonas oryzae* pv. Oryzae. Artif. Cells Nanomed. Biotechnol..

[23] Singh J., Kumar S., Alok A., Kumar Upadhyay S., Rawat M., Tsang D.C.W., Bolan N., Kim K.-H. (2019). The potential of green synthesized zinc oxide nanoparticles as nutrient source for plant growth. J. Clean. Prod..

[24] Verma R., Pathak S., Srivastava A.K., Prawer S., Tomljenovic-Hanic S. (2021). ZnO nanomaterials: Green synthesis, toxicity evaluation and new insights in biomedical applications. J. Alloys Compd..

[25] Mostafa A.M., Mwafy E.A., Toghan A. (2021). ZnO nanoparticles decorated carbon nanotubes via pulsed laser ablation method for degradation of methylene blue dyes. Colloids Surf. A Physicochem. Eng. Asp..

[26] Mohamed M.M., Ghanem M.A., Khairy M., Naguib E., Alotaibi N.H. (2019). Zinc oxide incorporated carbon nanotubes or graphene oxide nanohybrids for enhanced sonophotocatalytic degradation of methylene blue dye. Appl. Surf. Sci..

[27] Phin H.-Y., Ong Y.-T., Sin J.-C. (2020). Effect of carbon nanotubes loading on the photocatalytic activity of zinc oxide/carbon nanotubes photocatalyst synthesized via a modified sol-gel method. J. Environ. Chem. Eng..

[28] Hanif M.A., Kim Y.-S., Akter J., Kim H.G., Kwac L.K. (2023). Fabrication of Robust and Stable N-Doped ZnO/Single-Walled Carbon Nanotubes: Characterization, Photocatalytic Application, Kinetics, Degradation Products, and Toxicity Analysis. ACS Omega.

[29] Hanif M.A., Akter J., Lee I., Islam M.A., Sapkota K.P., Abbas H.G., Hahn J.R. (2021). Formation of chemical heterojunctions between ZnO nanoparticles and single-walled carbon nanotubes for synergistic enhancement of photocatalytic activity. J. Photochem. Photobiol. A Chem..

[30] Aljeboree A.M., Hussein S.A., Jawad M.A., Alkaim A.F. (2024). Hydrothermal synthesis of eco-friendly ZnO/CNT nanocomposite and efficient removal of Brilliant Green cationic dye. Results Chem..

[31] Oda A.M., Khudheyer F.Y., Obaid E.K. (2023). Photocatalytic Decolorization of Crystal Violate Dye Solution by ZnO/MWCNT Nanocomposite. Iraqi J. Sci..

[32] Pang Z., Sun X., Wu X., Nie Y., Liu Z., Yue L. (2015). Fabrication and application of carbon nanotubes/cellulose composite paper. Vacuum.

[33] Premanathan M., Karthikeyan K., Jeyasubramanian K., Manivannan G. (2011). Selective toxicity of ZnO nanoparticles toward Gram-positive bacteria and cancer cells by apoptosis through lipid peroxidation. Nanomed. Nanotechnol. Biol. Med..

[34] Manke A., Wang L., Rojanasakul Y. (2013). Mechanisms of Nanoparticle-Induced Oxidative Stress and Toxicity. BioMed Res. Int..

[35] Mammari N., Lamouroux E., Boudier A., Duval R.E. (2022). Current Knowledge on the Oxidative-Stress-Mediated Antimicrobial Properties of Metal-Based Nanoparticles. Microorganisms.

[36] Sirelkhatim A., Mahmud S., Seeni A., Kaus N.H.M., Ann L.C., Mohd Bakhori S.K., Hasan H., Mohamad D. (2015). Review on Zinc Oxide Nanoparticles: Antibacterial Activity and Toxicity Mechanism. Nano-Micro Lett..

[37] Mendes C.R., Dilarri G., Forsan C.F., Sapata V.D.M.R., Lopes P.R.M., Bueno de Moraes P., Montagnolli R.N., Ferreira H., Bidoia E.D. (2022). Antibacterial action and target mechanisms of zinc oxide nanoparticles against bacterial pathogens. Sci. Rep..

[38] Penchev H., Ublekov F., Budurova D., Sinigersky V. (2017). Novel electrospun polybenzimidazole fibers and yarns from ethanol/potassium hydroxide solution. Mater. Lett..

[39] Kraus W., Nolze G. (2000). PowderCell for Windows.

[40] Williams G.K., Hall W.H. (1953). X-ray line broadening from filed aluminium and wolfram. Acta Metall..

[41] Sarker S.D., Nahar L., Kumarasamy Y. (2007). Microtitre plate-based antibacterial assay incorporating resazurin as an indicator of cell growth, and its application in the in vitro antibacterial screening of phytochemicals. Methods.

[42] (2013). Standard Test Method for Determining the Antimicrobial Activity of Immobilized Antimicrobial Agents Under Dynamic Contact Conditions.

[43] Marra A., Silvestre C., Duraccio D., Cimmino S. (2016). Polylactic acid/zinc oxide biocomposite films for food packaging application. Int. J. Biol. Macromol..

[44] Kórösi L., Pertics B., Schneider G., Bognár B., Kovács J., Meynen V., Scarpellini A., Pasquale L., Prato M. (2020). Photocatalytic Inactivation of Plant Pathogenic Bacteria Using TiO_2_ Nanoparticles Prepared Hydrothermally. Nanomaterials.

[45] Kang J.-Y., Eo S.-M., Jeon I.-Y., Choi Y.S., Tan L.-S., Baek J.-B. (2010). Multifunctional Poly(2,5-benzimidazole)/Carbon Nanotube. J. Polym. Sci. Part A Polym. Chem..

[46] Eren E.O., Ozkan N., Devrim Y. (2021). Polybenzimidazole-modified carbon nanotubes as a support material for platinum-based hightemperature proton exchange membrane fuel cell electrocatalysts Composite Films. Int. J. Hydrogen Energy.

[47] Jung W.R., Choi J.H., Lee N., Shin K., Moon J.-H., Seo Y.-S. (2012). Reduced damage to carbon nanotubes during ultrasound-assisted dispersion as a result of supercritical-fluid treatment. Carbon.

[48] Rennhofer H., Zanghellini B. (2021). Dispersion State and Damage of Carbon Nanotubes and Carbon Nanofibers by Ultrasonic Dispersion: A Review. Nanomaterials.

[49] Penchev H., Zaharieva K., Milenova K., Ublekov F., Dimova S., Budurova D., Staneva M., Stambolova I., Sinigersky V., Blaskov V. (2018). Novel meta-and AB-polybenzimidazole/zinc oxide polymer hybrid nanomaterials for photocatalytic degradation of organic dyes. Mater. Lett..

[50] Badaire S., Poulin P., Maugey M., Zakri C. (2004). In situ measurements of nanotube dimensions in suspensions by depolarized dynamic light scattering. Langmuir.

[51] Malvern Instruments Limited (2015). Whitepaper, Characterization of Single Wall Carbon Nanotubes (SWNTs) by Combining Dynamic Light Scattering and Raman Spectroscopy.

[52] Kuzmenko V., Wang N., Haque M., Naboka O., Flygare M., Svensson K., Gatenholm P., Liu J., Enoksson P. (2017). Cellulose-derived carbon nanofibers/grapheme composite electrodes for powerful compact supercapacitors. RSC Adv..

[53] Xin L., Yang F., Qiu Y., Uzunoglu A., Rockward T., Borup R.L., Stanciu L.A., Li W., Xie J. (2016). Polybenzimidazole (PBI) Functionalized Nanographene as Highly Stable Catalyst Support for Polymer Electrolyte Membrane Fuel Cells (PEMFCs). J. Electrochem. Soc..

[54] Moraes R.A., Matos C.F., Castro E.G., Schreiner W.H., Oliveira M.M., Zarbin A.J.G. (2011). The Effect of Different Chemical Treatments on the Structure and Stability of Aqueous Dispersion of Iron- and Iron Oxide-Filled Multi-Walled Carbon Nanotubes. J. Braz. Chem. Soc..

[55] Bagabas A., Alshammari A., Aboud M.F., Kosslick H. (2013). Room-temperature synthesis of zinc oxide nanoparticles in different media and their application in cyanide photodegradation. Nanoscale Res. Lett..

[56] Bagheri M., Rostami Najafabadi N., Borna E. (2020). Removal of reactive blue 203 dye photocatalytic using ZnO nanoparticles stabilized on functionalized MWCNTs. J. King Saud Univ. Sci..

[57] Ahmad M., Ahmed E., Hong Z.L., Ahmed W., Elhissi A., Khalid N.R. (2014). Photocatalytic, sonocatalytic and sonophotocatalytic degradation of Rhodamine B using ZnO/CNTs composites photocatalysts. Ultrason. Sonochemistry.

[58] Amenu B., Taddesse A.M., Kebede T., Mengesha E.T., Bezu Z. (2024). Polyaniline-supported MWCNTs/ZnO/Ag_2_CO_3_ composite with enhanced photocatalytic and antimicrobial applications. Environ. Nanotechnol. Monit. Manag..

[59] Mohamed M.A., Abd Mutalib M., Mohd Hir Z.A., Zain M.F.M., Mohamad A.B., Minggu L.J., Awang N.A., Salleh W.N.W. (2017). An overview on cellulose-based material in tailoring bio-hybrid nanostructured photocatalysts for water treatment and renewable energy applications. Int. J. Biol. Macromol..

[60] Bu I.Y.Y. (2014). Optoelectronic properties of solution synthesis of carbon nanotube/ZnO:Al:N nanocomposite and its potential as a photocatalyst. Mater. Sci. Semicond. Process..

[61] Dostanić J.M., Lončarević D.R., Banković P.T., Cvetković O.G., Jovanović D.M., Mijin D.Ž. (2011). Influence of process parameters on the photodegradation of synthesized azo pyridone dye in TiO_2_ water suspension under simulated sunlight. J. Environ. Sci. Health Part A Toxic/Hazard. Subst. Environ. Eng..

[62] Klink S., Keller A.B., Wild A.J., Baumert V.L., Gube M., Lehndorff E., Meyer N., Mueller C.W., Phillips R.P., Pausch J. (2022). Stable isotopes reveal that fungal residues contribute more to mineral-associated organic matter pools than plant residues. Soil Biol. Biochem..

[63] Kamaruzzaman N.F., Tan L.P., Hamdan R.H., Choong S.S., Wong W.K., Gibson A.J., Chivu A., de Fatima Pina M. (2019). Antimicrobial Polymers: The Potential Replacement of Existing Antibiotics?. Int. J. Mol. Sci..

[64] Kang S., Pinault M., Pfefferle L.D., Elimelech M. (2007). Single-walled carbon nanotubes exhibit strong antimicrobial activity. Langmuir.

[65] Alekish M., Ismail Z.B., Albiss B., Nawasrah S. (2018). In vitro antibacterial effects of zinc oxide nanoparticles on multiple drug-resistant strains of Staphylococcus aureus and Escherichia coli: An alternative approach for antibacterial therapy of mastitis in sheep. Vet. World.

[66] Maurer E.I., Comfort K.K., Hussain S.M., Schlager J.J., Mukhopadhyay S.M. (2012). Novel platform development using an assembly of carbon nanotube, nanogold and immobilized RNA capture element towards rapid, selective sensing of bacteria. Sensors.

